# Impact of sarcopenia on the prognosis and treatment of lung cancer: an umbrella review

**DOI:** 10.1007/s12672-022-00576-0

**Published:** 2022-10-28

**Authors:** Ting-Yu Lin, Yen-Fu Chen, Wei-Ting Wu, Der-Sheng Han, I.-Chen Tsai, Ke-Vin Chang, Levent Özçakar

**Affiliations:** 1 Department of Physical Medicine and Rehabilitation, Lo-Hsu Medical Foundation, Inc., Lotung Poh-Ai Hospital, Yilan, Taiwan; 2 Department of Internal Medicine, National Taiwan University Hospital, Yunlin Branch, Douliu, Yunlin Taiwan; 3 Department of Physical Medicine and Rehabilitation, National Taiwan University Hospital, College of Medicine, National Taiwan University, Taipei, Taiwan; 4 Department of Physical Medicine and Rehabilitation, National Taiwan University Hospital, Bei-Hu Branch, Taipei, Taiwan; 5 Institute of Clinical Medicine, National Yang Ming Chiao Tung University, Taipei, Taiwan; 6 Congenital Heart Disease Study Group, Asian Society of Cardiovascular Imaging, Seoul, Korea; 7 InnovaRad Inc., Taichung, Taiwan; 8 Center for Regional Anesthesia and Pain Medicine, Wang-Fang Hospital, Taipei Medical University, Taipei, Taiwan; 9 Department of Physical and Rehabilitation Medicine, Hacettepe University Medical School, Ankara, Turkey

**Keywords:** Muscle loss, Pulmonary, Malignancy, Frailty, Prognosis

## Abstract

**Background:**

Lung cancer is the leading cause of cancer-related mortality worldwide. Sarcopenia, defined as the loss of muscle mass and function, is known to cause adverse health outcomes. The purpose of this umbrella review was to integrate published systematic reviews and meta-analyses exploring sarcopenia and lung cancer to provide comprehensive knowledge on their relationship.

**Methods:**

Eligible studies were searched from scientific databases until June 28, 2022. Critical appraisal was performed using A Measurement Tool to Assess Systematic Reviews (AMSTAR) 2. The impact of sarcopenia on the pathophysiology, prevalence, and prognosis of lung cancer is summarized at the level of systematic reviews or meta-analyses.

**Results:**

Fourteen reviews and meta-analyses were conducted. The methodological quality was high for one review, low for nine, and critically low for four. The most common standard for diagnosing sarcopenia in the lung cancer population is computed tomography (CT) to measure the skeletal muscle index at the third lumbar vertebra (L3). Sarcopenia was highly prevalent among patients with lung cancer, with a pooled prevalence ranging from 42.8% to 45.0%. The association between sarcopenia and increased postoperative complications and decreased disease control rates with immune checkpoint inhibitors has been demonstrated. Mortality was significantly higher in sarcopenic patients than in non-sarcopenic patients with lung cancer, regardless of the stage of disease or type of treatment.

**Conclusions:**

Sarcopenia is a poor prognostic factor for lung cancer. Future studies are necessary to clarify the pathophysiology of sarcopenia and develop effective interventions for sarcopenia in patients with lung cancer.

**Supplementary Information:**

The online version contains supplementary material available at 10.1007/s12672-022-00576-0.

## Introduction

Lung cancer is a common and unfavorable type of malignancy [1]. Its incidence is on the rise globally, with more than two million estimated new cases per year [1]. The age-standardized cumulative lifetime risk is 3.80% for men and 1.77% for women, making it the second most prevalent cancer in both sexes [2, 3]. Surgical excision, chemotherapy, and radiotherapy have been the traditional cornerstones in the treatment, followed by targeted therapy and immunotherapy. The improved survival in industrialized countries is attributed to decline in tobacco smoking, early detection via low-dose chest tomography, and easy access to the state-of-the-art treatment modalities [3]. Despite substantial efforts and advances, the latest 5-year survival rate (from 2010 to 2016) for lung cancer in the United States of America is 20.5% [4].

Tumor/node/metastasis (TNM) staging based on tumor size, local invasion, and distant spread is the prevailing framework for estimating life expectancy in the cancer population [5]. However, the utility of the TNM system is limited in advanced cancer and in patients receiving targeted therapy and immunotherapy. The functional status represented by the Eastern Cooperative Oncology Group (ECOG) Performance Status Scale is of independent prognostic value in lung cancer. However, its clinical value is limited by its subjective assessment [6]. Weight loss at the initial diagnosis was independently associated with poor outcomes in patients with non-small cell lung cancer (NSCLC) and small cell lung cancer (SCLC) [7]. Further, patients with NSCLC and weight loss are less responsive to chemotherapy and have an increased withdrawal rate [8]. Therefore, numerous ongoing studies aim to identify more reliable prognostic indicators other than weight loss.

Sarcopenia is a skeletal muscle disorder characterized by progressive generalized loss of muscle mass and function [9, 10]. In case of low muscle strength, sarcopenia can be confirmed by measuring the muscle quantity and quality. Although it was first introduced as a geriatric disease, the condition is not exclusive to older adults and can accompany many diseases. Its associations with cardiac disease, respiratory disease, cognitive impairment, and musculoskeletal disorders have also been observed [11, 12]. Sarcopenia is a pressing clinical issue because it poses increased risks for falls, fractures, functional impairment, hospitalizations, and mortality, and creates hefty healthcare burdens [11, 13]. Accordingly, there has been great interest in the impact of sarcopenia on lung cancer, with several systematic reviews and meta-analyses published to explore the relationship between them. This umbrella review aimed to compile evidence from these systematic reviews and meta-analyses to evaluate the existing information on the interplay between sarcopenia and lung cancer.

## Methods

### Protocol registration

We conducted an umbrella review according to the guidelines of Preferred Reporting Items for Systematic Reviews and Meta-Analysis (PRISMA) [14]. The protocol was registered at Inplasy.com with the number INPLASY202270050.

### Search strategy

PubMed, Embase, Web of Science, and Cochrane databases were systematically searched from their inception to June 2022 for articles assessing the relationship between sarcopenia and lung cancer. The following search terms were used: (“cancer” OR “lung cancer” OR “lung neoplasm” OR “lung tumor”) AND (“sarcopenia” OR “skeletal muscle” OR “muscle loss” OR “nutrition”) AND (“systematic review” OR “meta-analysis”). No language restrictions were applied. The gray literature was explored using Google Scholar. Furthermore, the reference lists of eligible articles were manually searched for additional relevant studies. The complete search strategy is presented in the Additional file 1.

### Inclusion and exclusion criteria

Articles were included if they (1) were systematic reviews or meta-analyses and (2) investigated the prevalence, pathophysiology, prognostic capability, or management of sarcopenia in patients with lung cancer. We excluded articles that (1) failed to complete a systematic literature search, (2) did not incorporate participants with primary pulmonary malignancy, (3) analyzed tumors from multiple sites but did not focus on or did not perform a subgroup analysis on lung cancer, and (4) only reported nutritional assessment without defining whether the patients were sarcopenic. Scoping reviews, narrative reviews, review protocols, and conference abstracts were excluded.

### Article selection and data extraction

The titles and abstracts of the papers retrieved from the initial database search were independently screened by two authors (T.-Y. L. and W.-T.W.). The full texts of each potential piece were obtained and reviewed for data extraction. The collected information encompassed the following: first author, country/year of publication, number of included studies/participants, aim of the review, diagnostic criteria for sarcopenia, qualitative outcomes, results from quantitative analyses (effect size, effect model, 95% confidence interval [CI], *p* value, and I^2^), and major findings. Any disagreement regarding study selection or data extraction was settled by the corresponding author.

### Quality assessment

Two authors (T.-Y. L. and W.-T.W.) separately performed the critical appraisal of included reviews using A Measurement Tool to Assess Systematic Reviews (AMSTAR) 2, and a consensus was reached after discussion [15]. AMSTAR2, a methodological quality evaluation tool, comprises 16 items, seven of which are of particular importance, i.e., the presence of a precedent protocol, comprehensive literature search, written inclusion/exclusion criteria, risk of bias assessment, appropriate statistical method, sufficient data interpretation, and publication bias consideration. After scoring yes, partial yes, or no for each item, the overall confidence of the systematic review or meta-analysis was graded as high, moderate, low, or critically low.

### Data synthesis

The results of this umbrella review are presented at the systematic review or meta-analysis level. We addressed the similarities and differences in the population, criteria for sarcopenia, and relevant outcomes to gain a complete understanding of the association between sarcopenia and lung cancer. Details of the studies included in each of the eligible reviews are outlined in the Additional file 1.

## Results

### Literature search

**Fig. 1:**
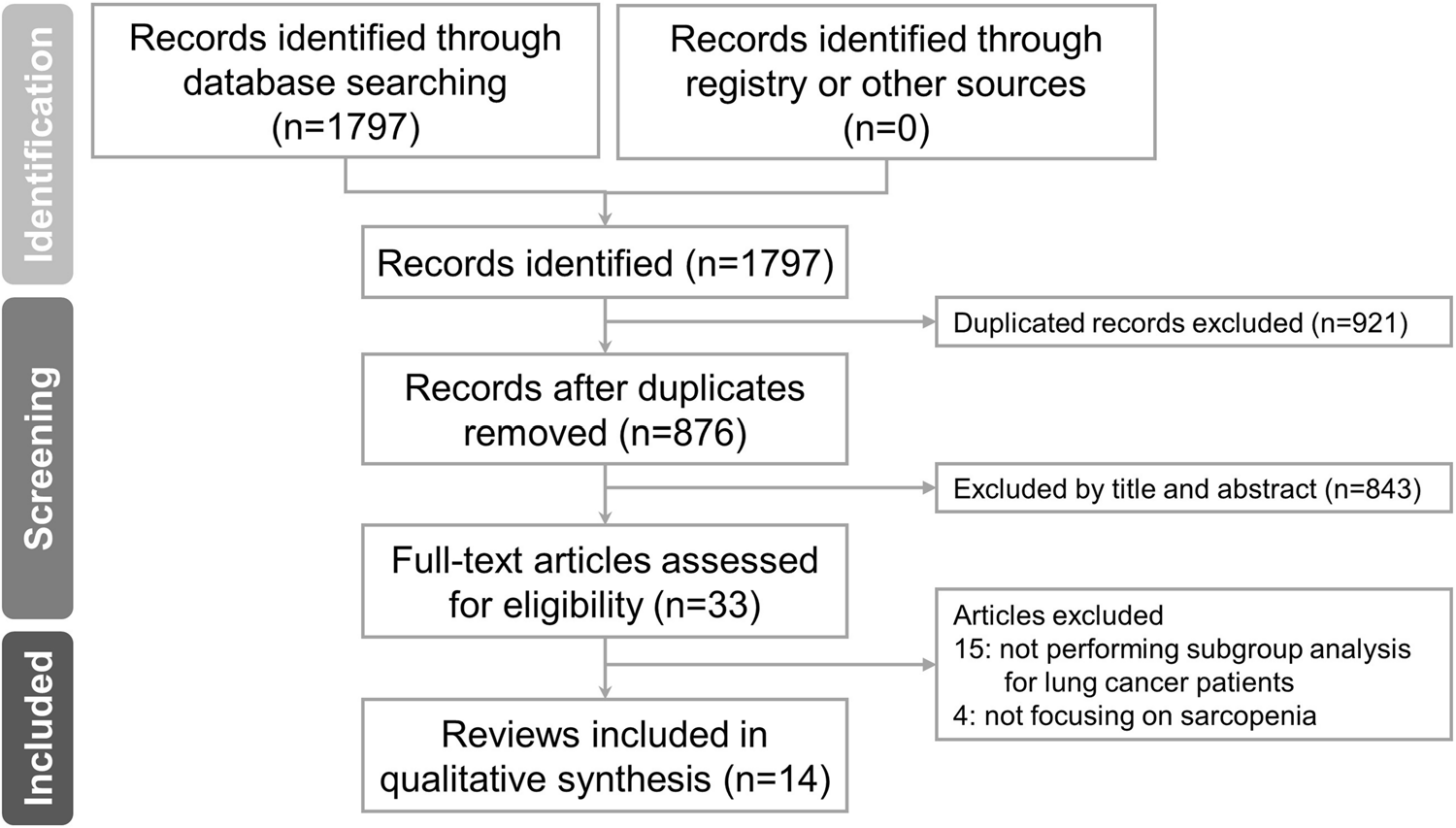
Flow diagram for the literature search

Of the 1797 records generated from the original database search, 1764 were removed for being duplicates or non-relevant literature after title and abstract screening. Full texts were screened for the remaining 33 articles; 15 articles were excluded wherein patients with lung cancer were not regarded as a subgroup during analysis and four for describing nutritional status without focusing on sarcopenia. Finally, 14 reviews [16–29] fulfilled all eligibility criteria and were included in our umbrella review (Fig. 1).

### Study characteristics

Nearly all reviews (except for one [16]) were published after 2019. The research team of eight [18, 20–25, 27] reviews was based in Asian countries, five [16, 17, 26, 28, 29] in European countries, and one [19] in the United States of America. Eight [16–21, 24, 29] reviews focused on malignancies of pulmonary origin, with four [18, 21, 24, 29] solely examining NSCLC and four [16, 17, 19, 20] encompassing all lung cancers. The remaining six [22, 23, 25–28] reviews incorporated tumors from multiple locations and dedicated a portion to lung cancer. The majority of reviews revealed the impact of sarcopenia on short-and long-term outcomes in the lung cancer population. Two [16, 29] focused on the broad scope of nutritional status evaluation, and two [26, 28] were centered on the prevalence of sarcopenia. Meta-analyses were conducted in eleven publications [17–25, 27, 28]. The characteristics of the included reviews are outlined in Table 1.

**Table 1:** Characteristics of the included reviews

Author, Year	Country	Protocol registration	Included studies (n)	Patients (n)	Searched database	Cancer population	Anticancer treatment	Research question
Collins et al., 2013	UK	No	35	4969 in total, over 2275 lung cancer	MEDLINE, MEDLINE In-Process, Embase, AMED, Cochrane	All stages of lung cancer	Surgery, chemotherapy (most studies not focusing on anticancer treatment)	To understand the relationship between muscle mass, muscle function and lung cancer
Buentzel et al., 2019	Germany	No	15	2521	PubMed, BioMed Central, Science Direct, Cochrane	All stages of lung cancer	Surgery, chemotherapy, radiotherapy, immunotherapy, combination	To investigate the influence of sarcopenia on the prognosis of lung cancer
Deng et al., 2019	China	No	6	1213	PubMed, Embase, Cochrane	All stages of NSCLC	Surgery	To investigate the impact of sarcopenia on survival of patients with surgically treated NSCLC
Nishimura et al., 2019	USA	No	9	1661	PubMed	All stages of lung cancer	Surgery	To determine the prevalence of sarcopenia in patients undergoing surgery for lung cancer and evaluate the prognostic impact of CT-defined sarcopenia
Yang et al., 2019	China	No	13	1880	MEDLINE, Embase, Cochrane CENTRAL	All stages of lung cancer	Surgery, chemotherapy, radiotherapy, immunotherapy, supportive care, combination	To summarize evidence on the prognostic role of sarcopenia in lung cancer patients
Wang et al., 2020	China	No	9	576	PubMed, Embase, Cochrane CENTRAL, ASCO, ESMO	Advanced, metastatic, recurrent NSCLC	Immunotherapy	To evaluate the association between pre-treatment sarcopenia and outcomes of immunotherapy in NSCLC patients
Au et al., 2021	Hong Kong, Taiwan	No	100 in total, 5 in the lung cancer subgroup	806 in the lung cancer subgroup	PubMed, Cochrane, Embase	All cancer types	Not reported	To evaluate the role of lean mass on mortality in different types of cancer
Deng et al., 2021	China	No	9 in total, 5 in the NSCLC subgroup	740 in total, 236 in the NSCLC subgroup	Web of Science, PubMed, Embase	Advanced cancers	Immunotherapy	To determine the prognostic ability of sarcopenia for advanced cancer patients treated with immune checkpoint inhibitors
Kawaguchi et al., 2021	Japan	No	10	2643	PubMed	All stages of NSCLC	Surgery	To investigate the predictive ability of sarcopenia in lung cancer patients
Lee et al., 2021	Korea	No	14 in total, 6 in the NSCLC subgroup	1284 in total, 294 in the NSCLC subgroup	PubMed, Embase Cochrane	All cancer types	Immunotherapy	To evaluate the long-term effects of sarcopenia on cancer patients on immune checkpoint inhibitors
Mcgovern et al., 2021	UK	No	160 in total, 11 in the NSCLC subgroup	42,063 in total, 2401 NSCLC patients	PubMed	Solid cancers	Not reported	To assess the prevalence of low SMI and SMD in common solid cancers
Takenaka et al., 2021	Japan	Yes	26 in total, 8 in the NSCLC subgroup	2501 in total, 631 in the NSCLC subgroup	PubMed, Scopus, Ichushi-Web	Solid cancers	Immunotherapy	To evaluate the prognostic value of sarcopenia of cancer patients treated with immune checkpoint inhibitors
Surov et al., 2022	Germany	No	280 in total, 16 in the lung cancer subgroup	81,814 in total, 3187 in the lung cancer subgroup	MEDLINE, Cochrane, SCOPUS	Solid cancers	Not reported	To analyze the prevalence of low skeletal muscle mass in different solid malignancies
Voorn et al., 2022	Netherlands	Yes	23 in total, 9 evaluating sarcopenia	7522 in total, 1351 in the NSCLC subgroup	PubMed, Embase, Cinahl	Stage I-III NSCLC	Surgery, chemotherapy, radiotherapy	To evaluate the association between pre-treatment nutritional assessments and complication rates in patients with stage I-III NSCLC

*ASCO* American Society of Clinical Oncology, *SMI* skeletal muscle mass index, *SMD* skeletal muscle mass density, *ESMO* European Society of Medical Oncology, *NSCLC* non-small cell lung cancer

### Methodological quality of the included studies

According to the critical appraisal using AMSTAR2, one review [20] had high, nine had low [17, 18, 21–23, 25, 27–29], and four [16, 17, 19, 24, 26] had critically low evidence quality. Common methodological problems were lack of prior protocol registration (only registered in two reviews [27, 29]), not employing a comprehensive search strategy (three reviews [19, 24, 26] used only PubMed for literature search and 12 [16–19, 22–29] did not consider trial registries), and not providing sufficient information on the excluded studies (only recorded in three reviews [16, 20, 25]). Seven reviews [18, 19, 22–24, 26, 28] did not elaborate on duplicate study selection, and seven [16, 17, 22, 23, 26–28] did not show independent data extraction by two authors. The checklist is presented in Table 2.

**Table 2:** AMSTAR-2 checklist

Author, year	AMSTAR-2 Item Number															
	1	2	3	4	5	6	7	8	9	10	11	12	13	14	15	16
Collins et al., 2013	Y	N	Y	PY	Y	N	Y	PY	N	Y	N/A	N/A	Y	Y	N/A	Y
Buentzel et al., 2019	Y	PY	Y	PY	Y	N	N	PY	Y	Y	Y	Y	Y	Y	Y	Y
Deng et al., 2019	Y	PY	Y	PY	N	Y	N	Y	Y	Y	Y	Y	Y	Y	Y	Y
Nishimura et al., 2019	Y	PY	Y	N	N	Y	N	Y	Y	Y	Y	Y	Y	Y	N	N
Yang et al., 2019	Y	PY	Y	Y	Y	Y	Y	Y	Y	Y	Y	Y	Y	Y	Y	Y
Wang et al., 2020	Y	PY	Y	Y	Y	Y	N	PY	Y	Y	Y	Y	Y	Y	Y	Y
Au et al., 2021	Y	PY	Y	PY	N	N	N	N	Y	Y	Y	Y	Y	Y	Y	Y
Deng et al., 2021	Y	PY	Y	PY	N	N	N	Y	Y	Y	Y	Y	Y	Y	Y	Y
Kawaguchi et al., 2021	Y	PY	Y	N	N	Y	N	Y	Y	Y	Y	N	Y	Y	Y	Y
Lee et al., 2021	Y	PY	Y	PY	Y	Y	Y	Y	Y	Y	Y	Y	Y	Y	N	Y
McGovern, 2021	Y	PY	Y	N	N	N	N	N	Y	Y	N/A	N/A	Y	Y	N/A	Y
Takenaka et al., 2021	Y	Y	Y	PY	Y	N	N	PY	Y	Y	Y	Y	Y	Y	Y	Y
Surov et al., 2022	Y	PY	Y	PY	N	N	N	N	Y	Y	Y	Y	Y	Y	Y	Y
Voorm et al., 2022	Y	Y	Y	PY	Y	Y	N	N	Y	Y	N/A	N/A	Y	Y	N/A	Y

*Y* yes, *N* no, *PY* partial yes, *N/A* not applicable due to absence of meta-analyses. 1 = PICO Elements; 2 = Prior Protocol; 3 = Study Designs; 4 = Search Strategy; 5 = Study Selection; 6 = Data Extraction; 7 = Excluded Studies; 8 = PICO Details; 9 = Risk of Bias Assessment; 10 Funding Sources; 11 = Meta-Analysis Methods; 12 = Risk of Bias Impact on Results; 13 = Risk of Bias Discussion; 14 = Explain Heterogeneity; 15 = Publication Bias; 16 = Conflict of Interest

### Diagnosis and prevalence of sarcopenia in lung cancer

In a pioneer review by Collins et al. [16], sarcopenia was defined by a handful of measuring techniques, including dual-energy x-ray absorptiometry (DEXA), bioelectrical impedance analysis (BIA), computed tomography (CT), upper arm dimensions, grip strength, and skinfold thickness. More recently, CT has become the dominant tool for confirming the diagnosis (Table 3). Skeletal muscle index (SMI, cm^2^/m^2^) is calculated by dividing the cumulative skeletal muscle area (SMA, cm^2^) on a transverse CT slice by the square of the patient’s height. Psoas muscle index (PMI) is calculated using the following formula: total psoas muscle area (cm^2^) / height (m^2^). Skeletal muscle density (HU) is another indicator of body composition on CT images, reflecting the intramuscular adipose tissue infiltration or muscle quality. The systematic review by McGovern et al. [26] revealed that the most popular diagnostic thresholds for sarcopenia were derived from large-population studies by Prado et al. [30] (n = 250) and Martin et al. [31] (n = 1473).

**Table 3:** Results and main findings of the included reviews

Author, year	Skeletal muscle assessment and cutoff values	Meta-analysis	Main finding
Collins et al., 2013	1. DEXA: fat-free mass, fat-free mass index, lean body mass, appendicular lean mass, appendicular muscle mass index, body cell mass, body cell mass index, skeletal muscle mass index 2. BIA: fat-free mass, fat-free mass index, lean body mass, appendicular skeletal muscle mass, upper arm measurements 3. CT: SMA at L3 and T4, SMI at L3, mid-upper arm circumference, arm muscle area 4. Upper arm measurements 5. Grip strength 6. Four skinfold thickens 7. Tritium/deuterium dilution 8. Total body potassium Cutoff values were not presented in this review	Not performed	Sarcopenia was highly prevalent in patients with lung cancer and was associated with poorer functional status and overall survival. There was limited evidence on the correlation between muscle loss and muscle function. The mechanism of sarcopenia and effect of supplements among lung cancer patients were uncertain
Buentzel et al., 2019	1. DEXA: < 7.26 kg/m^2^ for men, < 5.45 kg/m^2^ for women 2. BIA: phase angle ≤ 5.3 or 5.8 degrees 3. CT: SMI at L3 < 41–55 cm^2^/m^2^ for men and < 38–41.10 cm^2^/m^2^ for women, SMI at T4 < 67.3 cm^2^/m^2^ for men and < 46.3 cm^2^/m^2^ for women, SMD < 28–44.1 HU for men and < 23.8–40.5 HU for women	Overall survival	Sarcopenia was an independent risk factor for death regardless of the stage of lung cancer. Multivariate meta-analysis showed a three-fold increased death risk in those with sarcopenia
Deng et al., 2019	CT: SMI at L3 < 43.75–55 cm^2^/m^2^ for men and < 39–41.10 cm^2^/m^2^ for women, PMI at L3 < 6.36 cm^2^/m^2^ for men and < 3.92 cm^2^/m^2^ for women, PMA at L3 ≤ 1601 mm^2^ for men and ≤ 999 mm^2^ for women	1. Overall survival 2. Disease free survival	NSCLC patients with sarcopenia had significantly lower 5-year overall survival, especially those with early-stage disease. No significant difference in 5-year disease free survival was found due to insufficient data
Nishimura et al., 2019	CT: SMI at L3 < 43.75–55 cm^2^/m^2^ for men and < 39–41.10 cm^2^/m^2^ for women, SMA at T5 < 181.2 cm^2^ for men and < 129.4 cm^2^ for women, SMA at T8 < 115.3 cm^2^ for men and < 74.0 cm^2^ for women, PMI at L3 < 6.36 cm^2^/m^2^ for men and < 3.92 cm^2^/m^2^ for women, PMA at L3 ≤ 1601 mm^2^ for men and ≤ 999 mm^2^ for women	1. Prevalence 2. Post-operative complications 3. Overall survival	The overall prevalence of sarcopenia was 42.8%. There was increased risk of early complications and worse survival in lung cancer patients with concomitant sarcopenia
Yang et al., 2019	1. DEXA: ALM < 7.26 for men and < 5.45 kg/m^2^ for women 2. CT: SMI at L3 < 41–55 cm^2^/m^2^ for men and < 33–41.10 cm^2^/m^2^ for women, SMI at T4 < 437 mm^2^/m^2^ for men, PMI at L3 < 6.36 cm^2^/m^2^ for men and < 3.92 cm^2^/m^2^for women, one side PMI < 2.93 cm^2^/m^2^ or < 2.4 cm^2^/m^2^ for women	1. Prevalence 2. Overall survival 3. Disease free survival	The pooled prevalence of sarcopenia was 45%. Sarcopenia was associated with shorter overall survival regardless of cancer type (NSCLC/SCLC) or stage
Wang et al., 2020	CT: SMI at L3 < 25.63–53 cm^2^/m^2^ for men and < 21.73–41 cm^2^/m^2^ for women, PMI at L3 < 6.36 cm^2^/m^2^ for men and < 3.92 cm^2^/m^2^ for women	1. Overall survival 2. Progression free survival 3. Disease free survival 4. Overall survival 5. Immune-related adverse events	Pre-immunotherapy sarcopenia and worsening sarcopenia during treatment was predictive of unfavorable overall survival, progression free survival and disease control rate for NSCLC patients. No significant association was observed regarding response rate or adverse events
Au et al., 2021	CT: SMI at L3 < 41–55 cm^2^/m^2^ for men and < 38–41.10 cm^2^/m^2^ for women, PMA at L3 ≤ 1601 mm^2^ for men and ≤ 999 mm^2^ for women	Mortality	Low lean mass was significantly associated with higher mortality in lung cancer
Deng et al., 2021	CT: SMI at L3 < 25.63–53 cm^2^/m^2^ for men and < 21.73–41 cm^2^/m^2^ for women, PMI at L3 < 6.36 cm^2^/m^2^ for men and < 3.92 cm^2^/m^2^ for women	1. Overall survival 2. Progression free survival	NSCLC patients with sarcopenia had significantly lower 1-year progression free survival and overall survival. Sarcopenic NSCLC patients also tended to have lower response rates
Kawaguchi et al., 2021	CT: SMI at L3 ≤ 43.75–55 cm^2^/m^2^ for men and < 38.5–41.10 cm^2^/m^2^ for women, PMI at L3 < 3.70–8.71 cm^2^/m^2^ for men and < 2.50–6.51 cm^2^/m^2^ for women, total muscular mass index < 6.49 kg/m^2^	1. Overall survival 2. Disease free survival 3. Post-operative complications	There were worse overall survival/disease free survival and more postoperative complications in NSCLC patients with sarcopenia
Lee et al., 2021	CT: SMI at L3 < 25.63 cm^2^/m^2^ for men and < 21.73 cm^2^/m^2^ for women, PMI at L3 < 6.36 cm^2^/m^2^ for men and < 3.92 cm^2^/m^2^ for women	1. Overall survival 2. Progression free survival	Sarcopenic cancer patients had poorer long-term prognosis. Progression free survival was significantly shorter in NSCLC patients treated with immune checkpoint inhibitors
Mcgovern et al., 2021	CT: SMI at L3 < 25.63–55 cm^2^/m^2^ for men and < 21.73–41.1 cm^2^/m^2^ for women, SMA at L3 < 135.8 cm^2^ for men and < 97.0 cm^2^ for women, SMD < 28–41 HU for men and < 23.8–41 HU for women	Not performed	The median percentage of lung cancer patients with low SMI and SMD was 49.5% and 19.3%, respectively
Takenaka et al., 2021	CT: SMI at L3 < 25.63–52.4 cm^2^/m^2^ for men and < 21.73–41 cm^2^/m^2^ for women, PMI at L3 < 6.36 cm^2^/m^2^ for men and < 3.92 cm^2^/m^2^ for women, SMD < 33–41 HU for men and women	1. Overall survival 2. Progression free survival 3. Disease free survival 4. Overall survival	Sarcopenia was a predictor for worse overall survival, disease free survival and disease control rate in NSCLC patients. Sarcopenia was not associated with severe adverse events in solid cancer patients on immune checkpoint inhibitors
Surov et al., 2022	CT: SMI at L3 < 43–55 cm^2^/m^2^ for men and < 38.5–41.10 cm^2^/m^2^ for women, SMI at L1 < 38 cm^2^/m^2^ for men and < 29.6 cm^2^/m^2^ for women, PMI at L3 < 6.36 cm^2^/m^2^ for men and < 3.92 cm^2^/m^2^ for women, psoas volume index 71.31 cm^3^/m^3^ for men and < 51.87 cm^3^/m^3^ for women	Prevalence	The overall prevalence of sarcopenia was 44% among lung cancer patients. Meanwhile, the prevalence was 36% and 51.5% in curative and palliative settings, respectively
Vroom et al., 2022	CT: SMI at L3 < 49–55 cm^2^/m^2^ for men and < 39 cm^2^/m^2^ for women, PMI at L3 < 3.70–6.36 cm^2^/m^2^ for men and < 2.50–3.92 cm^2^/m^2^ for women	Not performed	Sarcopenia was associated with higher risks of postoperative complications for lung cancer patients. The cutoff values for defining sarcopenia were inconsistent

*BIA* bioelectrical impedance analysis, *BMI* body mass index, *CT* computed tomography, *DEXA* dual-energy x-ray absorptiometry

*L1/3* first/third lumbar vertebrae, *NSCLC* non-small cell lung cancer, *PMA* psoas muscle area, *PMI* psoas muscle index, *SMA* skeletal muscle area, *SMI* skeletal muscle index,

*T4/5/8* fourth/fifth/eighth thoracic vertebrae. Note: Only the definitions used in the lung cancer subgroup were extracted from the reviews involving multiple tumor types

**Table 4:** Quantitative outcomes of the included meta-analyses

Outcome	Author, year	Population	Included studies (n)	Metric	Effects model	Effect size (95% CI)	p value	I^2^
Overall response rate	Wang et al., 2020	NSCLC treated with ICIs	6	RR	REM	0.54 (0.19–1.53)	N/A	57.4%
	Takenaka et al., 2021	NSCLC treated with ICIs	7	OR	REM	0.49 (0.20–1.22)	0.127	N/A
Disease control rate	Wang et al., 2020	NSCLC treated with ICIs	6	RR	FEM	0.70 (0.56–0.86)	0.001	38.1%
	Takenaka et al., 2021	NSCLC treated with ICIs	6	OR	REM	0.43 (0.22–0.87)	0.019	N/A
Post-operative complications	Nishumura et al., 2019	Operated lung cancer	4	OR	REM	2.51 (1.55–4.08)	0.0002	15%
	Kawaguchi et al., 2021	Operated NSCLC	6	OR	FEM	1.86 (1.42–2.44)	< 0.00001	60%
Immune-related adverse events	Wang et al., 2020	NSCLC treated with ICIs	3	RR	REM	0.99 (0.21–4.67)	0.986	61.8%
Disease free survival	Yang et al., 2019	NSCLC	3	Hazard ratio	REM	1.28 (0.44–3.69)	N/A	72%
	Deng et al., 2019	Operated NSCLC	3	RR	REM	1.14 (0.59–2.17)	0.70	72.1%
	Deng et al., 2019	Operated stage I NSCLC	2	RR	FEM	1.59 (1.01–2.52)	0.046	0.0%
	Kawaguchi et al., 2021	Operated NSCLC	6	OR	REM	1.66 (1.00–2.74)	0.05	63%
	Wang et al., 2020	NSCLC treated with ICIs	7	Hazard ratio	REM	1.98 (1.32–2.97)	0.001	53.3%
Progression free survival	Wang et al., 2020	NSCLC treated with ICIs	7	Hazard ratio	REM	1.98 (1.32–2.97)	0.046	53.3%
	Wang et al., 2020	NSCLC treated with ICIs	4	Hazard ratio (multivariate)	REM	1.77 (1.08–2.90)	N/A	57.7%
	Wang et al., 2020	NSCLC treated with nivolumab	2	Hazard ratio	FEM	2.59 (1.41–4.78)	N/A	0.0%
	Deng et al., 2021	NSCLC treated with ICIs	5	RR	FEM	1.39 (1.21–1.60)	< 0.001	0.0%
	Deng et al., 2021	NSCLC treated with ICIs	3	Hazard ratio (multivariate)	FEM	1.87 (1.30–2.68)	0.001	0.0%
	Lee et al., 2021	NSCLC treated with ICIs	3	Hazard ratio (multivariate)	REM	2.23 (1.58–3.15)	< 0.00001	0%
	Takenaka et al., 2021	NSCLC treated with ICIs	8	Hazard ratio	REM	1.69 (1.24–2.31)	0.001	N/A
Overall survival	Buentzel et al., 2019	Lung cancer	9	Hazard ratio	REM	1.96 (1.49–2.59)	< 0.0001	36%
	Buentzel et al., 2019	Lung cancer	7	Hazard ratio (multivariate)	REM	3.13 (2.06–4.76)	< 0.00001	37%
	Yang et al., 2019	Lung cancer	11	Hazard ratio	REM	2.23 (1.68–2.94)	N/A	50%
	Au et al., 2021	Lung cancer	5	HR	REM	2.19 (1.28–3.75)	0.004	60%
	Yang et al., 2019	NSCLC	9	Hazard ratio	REM	2.57 (1.79–3.68)	N/A	53%
	Yang et al., 2019	SCLC	2	Hazard ratio	FEM	1.59 (1.17–2.14)	N/A	0%
	Yang et al., 2019	Stage I-II NSCLC	5	Hazard ratio	REM	3.23 (1.68–6.23)	N/A	76%
	Buentzel et al., 2019	Stage I-IIIA NSCLC	3	Hazard ratio	REM	3.09 (1.75–5.46)	< 0.0001	0%
	Yang et al., 2019	Stage III-IV NSCLC	2	Hazard ratio	FEM	2.19 (1.14–4.24)	N/A	0%
	Buentzel et al., 2019	Stage IIIB-IV NSCLC	3	Hazard ratio	REM	2.38 (1.47–3.86)	0.0004	3%
	Deng et al., 2019	Operated NSCLC	6	RR	REM	1.63 (1.13–2.33)	0.008	73.1%
	Deng et al., 2019	Operated NSCLC	5	Hazard ratio	REM	2.85 (1.67–4.86)	< 0.001	64.5%
	Nishumura et al., 2019	Operated NSCLC	3	Hazard ratio	REM	2.31 (1.26–4.24)	0.007	69%
	Kawaguchi, 2021	Operated NSCLC	9	OR	FEM	3.07 (2.45–3.85)	< 0.00001	74%
	Deng et al., 2019	Operated Stage I NSCLC	3	RR	FEM	2.09 (1.51–2.88)	< 0.001	0.0%
	Deng et al., 2019	Operated Stage I NSCLC	3	Hazard ratio	FEM	4.68 (2.76–7.94)	< 0.001	0.0%
	Wang et al., 2020	NSCLC treated with ICIs	6	Hazard ratio	FEM	1.61 (1.24–2.10)	< 0.001	0.0%
	Wang et al., 2020	NSCLC treated with ICIs	3	Hazard ratio (multivariate)	FEM	1.60 (1.13–2.26)	N/A	0.0%
	Wang et al., 2020	NSCLC treated with nivolumab	3	Hazard ratio	FEM	2.10 (1.22–3.61)	N/A	0.0%
	Deng et al., 2021	NSCLC treated with ICIs	3	RR	FEM	2.15 (1.42–3.25)	< 0.001	0.0%
	Deng et al., 2021	NSCLC treated with ICIs	2	Hazard ratio (multivariate)	FEM	2.18 (1.33–3.56)	0.002	0.0%
	Lee et al., 2021	NSCLC treated with ICIs	3	Hazard ratio (multivariate)	REM	1.27 (0.71–2.26)	0.43	49%
	Takenaka et al., 2021	NSCLC treated with ICIs	6	Hazard ratio	REM	1.61 (1.19–2.18)	0.002	N/A

*FEM* fixed effect model, *ICI* immune checkpoint inhibitor, *N/A* not available, *NSCLC* non-small cell lung cancer, *OR* odds ratio, *REM* random effect model, *RR* risk ratio, *SCLC* small cell lung cancer

Four reviews [19, 20, 26, 28] investigated the prevalence of sarcopenia in patients with lung cancer. Yang et al. [20] recruited 1810 patients from 13 studies and concluded that the pooled prevalence was 45% (95% CI: 32–57%). Meanwhile, the prevalence was 43% (95% CI: 32–54%) in patients with NSCLC and 52% (95% CI: 32–57%) in patients with SCLC. Male patients were more sarcopenic than female patients (53.4% vs. 46.6% for NSCLC and 67.8% vs. 32.2% for SCLC). A review by Nishimura et al. [19] showed similar results, with an overall prevalence of 42.8%. McGovern et al. [26] assembled several studies on CT-assessed muscle mass in the cancer population, among which nine [32–40] measured SMI in patients with lung cancer and reported that the median percentage of patients with low SMI was 49.5%. Considering the extent of tumor growth, individuals with non-curative diseases (stage IV, unresectable, and metastatic lung cancer) suffered more frequently from muscle depletion than those with curative diseases (stage I–III lung cancer). The median percentage of sarcopenic patients was 50.3% in the former group compared to 40.2% in the latter group. Likewise, Surov et al. [28] revealed 44.0% regarding the summarized prevalence of sarcopenia from 16 studies, with 36.0% in curative and 51.1% in palliative settings.

### Pathophysiology and treatment of sarcopenia in lung cancer

Systematic reviews by Collins et al. [16] and Nishimura et al. [19] reported concurrent loss of body weight and muscle mass in patients with lung cancer. No significant difference in preoperative serum albumin was noted between the sarcopenic and non-sarcopenic groups [19]. Besides, carcinoembryonic antigen was associated with preoperative sarcopenia in a few, but not all studies, according to the review by Nishimura et al. [19]. Herein, there was no robust evidence on how changes in protein metabolism or genetic polymorphism contributed to the development of muscle mass loss in the lung cancer population [16].

Among the epidemiological variables, Kawaguchi et al. [24] reported a significant association between sarcopenia and smoking habits in three [41–43] out of five studies [33, 41–44]. Nishumura et al. [19] observed that aging was significantly associated with sarcopenia in half of the recruited studies while there was no significant association between sarcopenia and pathologic staging of lung cancer.

Regarding epidemiological variables, Kawaguchi et al. [24] discovered three [41–43] out of five [33, 41–44] studies reporting a significant association between sarcopenia and smoking habits. Nishumura et al. [19]’s review revealed that aging was significantly associated with sarcopenia in half of the selected studies, while there was no significant association between sarcopenia and the pathologic staging of lung cancer.

The performance status was not related to preoperative sarcopenia in the review by Nishumura et al. [19]. In contrast, Collins et al. [16] reported that patients with cachexia had a reduced walking distance and quadriceps strength. There are incoherent findings about the association between forced expiratory volume and preoperative sarcopenia [19]. The effect of nutritional supplements (such as fish oil, protein supplement, and adenosine-5'-triphosphate infusion) on slowing/reversing muscle loss or on improving survival in patients with lung cancer has been contradictory [16].

### The impact of sarcopenia on the prognosis of lung cancer

The prognostic value of sarcopenia in patients with lung cancer was fundamental in the majority of the included reviews. Four reviews [17, 20, 22, 29] incorporated diverse treatment options, such as surgery, chemotherapy, immunotherapy, radiotherapy, or palliative care. Three reviews [18, 19, 24] emphasized on the postoperative outcomes and four [21, 23, 25, 27] focused on immunotherapy.

#### Postoperative complication rate

The postoperative complication rate was increased in patients with sarcopenia with an odds ratio (OR) of 2.51 (95% CI: 1.55–4.08) in the meta-analysis by Nishumura et al. [19] (involving NSCLC, SCLC, and metastatic disease to the lung) and 1.86 (95% CI: 1.42–2.44) in that of Kawaguchi et al. [24] (targeting NSCLC). Additionally, two reviews [19, 24] reported that sarcopenic patients were more likely to withstand major complications according to a single study on 328 patients with NSCLC (16.1% vs. 7.1%, *p* = 0.036) [42]. Lower the SMI/PMI threshold for diagnosing sarcopenia, higher was the risk of enduring postoperative complications in NSCLC [24].

#### Overall response rate

The overall response rate refers to the percentage of patients whose tumors disappear (complete response) or decrease in size (partial response) after treatment. The disease control rate describes the proportion of patients with decreased or stable disease burden during the study period [45]. The endpoints in patients with NSCLC receiving immunotherapy were condensed in two meta-analyses [21, 27]; they revealed a significantly worse disease control rate in sarcopenic versus non-sarcopenic participants. Pre-treatment sarcopenia and deteriorating sarcopenic status after initiating therapies were linked to a decreased disease control rate (risk ratio [RR]: 0.62, 95% CI: 0.19–1.53) [21]. However, although sarcopenia showed an unfavorable overall response rate (RR: 0.54, 95% CI: 0.19–0.53), the difference between sarcopenic and non-sarcopenic patients was not statistically significant. Interestingly, a pooled result from three studies [38, 46, 47] suggested that sarcopenia did not increase the rate of immune-related adverse events (RR: 0.99, 95% CI: 0.21–4.67) such as dermatitis, colitis, pneumonitis, or endocrinopathies [21].

#### Progression-free survival

Progression-free survival implies the time before the detection of disease progression or patient’s death [48]. The duration without tumor relapse after treatment is represented by disease-free survival [49]. Pre-treatment sarcopenia was significantly related to shortened progression-free survival rates in patients with lung cancer receiving immunotherapy in the meta-analyses by Wang et al. [21], Deng et al. [23], Lee et al. [25] and Takenaka et al. [27]. The association of sarcopenia with disease-free survival varied among different patient populations. Deng et al. [18] and Yang et al. [20] did not acknowledge a significant difference in the postoperative disease-free survival between sarcopenic and non-sarcopenic patients with NSCLC [18, 20]. However, Kawaguchi et al.[24] reported that patients with NSCLC and sarcopenia had reduced disease-free survival after lung resections (OR: 1.66, 95% CI: 1.00–2.74). Poorer disease-free survival was also observed in sarcopenic patients with advanced NSCLC on immune checkpoint inhibitors (ICIs) with a hazard ratio (HR) of 1.98 (95% CI: 1.32–2.97) [21].

#### Overall survival

Patients with lung cancer and concomitant sarcopenia had worse overall survival than non-sarcopenic patients as demonstrated repeatedly in our umbrella review. Across various meta-analyses, the pooled HR and RR of mortality for sarcopenic patients ranged between 1.27–4.68 and 1.63–2.15, respectively [17–25, 27]. Buentzel et al. [17] studied patients with lung cancer receiving diverse anti-cancer therapies (surgery, targeted therapy, chemotherapy, radiotherapy, or a combination) and discovered that sarcopenia was an independent risk factor for mortality (HR: 3.13, 95% CI: 2.06–4.76). From 11 studies, Yang et al. [20] combined data from 1,621 patients with lung cancer and illustrated a significantly worse overall survival for those with sarcopenia (HR: 2.23, 95% CI: 1.68–2.94) than those without. Further subgroup analysis did not reveal any discrepancy by tumor type (NSCLC vs. SCLC) or staging (stage I–II vs. stage III–IV).

Regarding cancer treatment, sarcopenia was significantly associated with poor overall survival among either operated [18, 19, 24] or immunotherapy-managed [21, 23, 27] patients with NSCLC. Wang et al. [21] showed that sarcopenia was an independent unfavorable prognostic factor for patients with NSCLC on ICIs with an HR of 1.61(95% CI: 1.24–2.10) and it indicated higher mortality for the subgroup using nivolumab (HR: 2.10, 95% CI: 1.22–3.61). Buentzel et al. [17] and Yang et al. [20] reported that the cancer stage did not affect the predictability of sarcopenia for mortality. Deng et al. [18] noted that this was especially true for patients with stage I disease. In their meta-analysis, sarcopenia led to significantly poorer overall survival in patients with stage I NSCLC (RR: 2.09, 95% CI: 1.51–2.88). However, the correlation was not significant when studies recruiting NSCLC patients of all stages were analyzed (RR: 1.37, 95% CI: 0.78–2.42) [18]. For every one unit fall in SMA and SMI or for a one-degree decrease in the phase angle by BIA during the treatment for lung cancer, a 4% increase in mortality was observed [17]. Wang et al. [21] also observed that the presence of muscle loss under immunotherapy was predictive for poor overall survival (HR: 4.97, 95% CI: 2.39–10.32). Nonetheless, there were inconsistent findings regarding the median overall survival [20]. Sarcopenic patients had significantly poorer median overall survival than non-sarcopenic patients in SCLC (8.6 vs. 16.8 months, *p* = 0.031) [50], stage I NSCLC (32 vs. 112 months, *p* < 0.01) [51] and stage IV NSCLC (12.6 vs. 23.5 months, *p* = 0.035) [52] cohorts. However, the difference was not significant in stage IIIB–IV NSCLC (7.5 vs. 7.9 months, *p* = 0.490) [32] (Table 4).

## Discussion

**Fig. 2:**
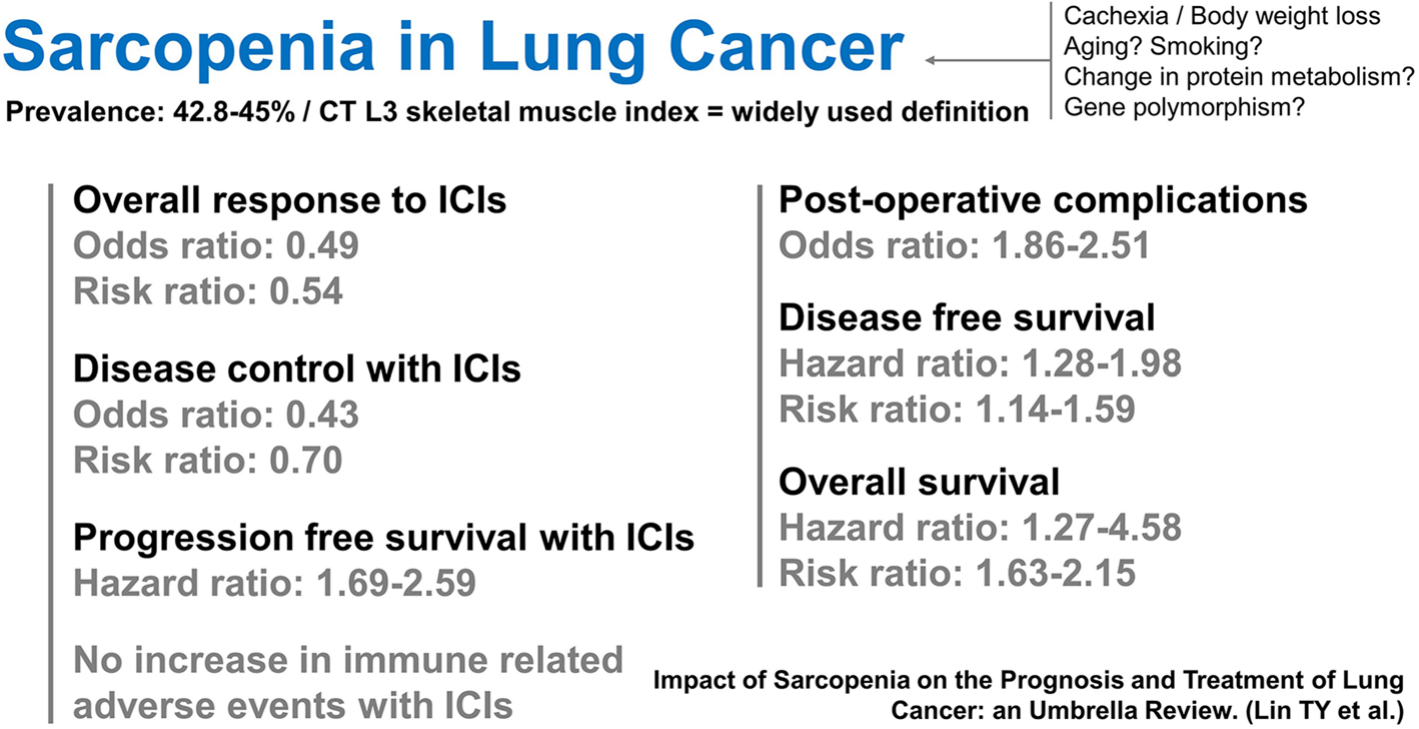
Summary of the findings of this umbrella review

According to this umbrella review, sarcopenia was prevalent among patients with lung cancer and served as an unfavorable prognostic factor. Similarly, sarcopenia was significantly associated with higher postoperative complications, lower disease control rates in patients using ICIs, and poorer overall survival. However, it does not increase the risk of immune-related side effects in patients receiving ICIs for lung cancer. The predictive value of sarcopenia for increased mortality remained unchanged across patients with different tumor types or those using distinct anti-cancer therapies. The findings of this umbrella review are summarized in Fig. 2.

We highlighted the pervasiveness of muscle depletion in lung cancer, with an overall prevalence of sarcopenia ranging from 42.8 to 45.0%. More patients with advanced disease were sarcopenic than those with early stage lung cancer. Sarcopenia is a part of the multifactorial cachexia syndrome. Patients suffering from cachexia experience profound body weight loss, primarily from the wasting of skeletal muscle and adipose tissue, anemia, and extra-cellular fluid imbalance [53]. The prevalence of cachexia ranges from 36 to 61% in NSCLC [54–56]. Anorexia, accelerated resting energy expenditure, increased lipolysis, and depression of protein synthesis coupled with rising protein degradation play a role in the development of cachexia [57]. Herein, although cachexic patients are known to be sarcopenic, the majority of sarcopenic people may not be cachexic [58]. Changes in the intertwined epigenic, cellular, and hormonal pathways of skeletal muscle metabolism that induce sarcopenia are not yet fully understood [11]. Immobility and insufficient calorie intake are the primary driving causes [59]. In patients with lung cancer, malignancy related pain, fatigue, and depression could lead to disuse atrophy. The side effects of antineoplastic therapy, such as nausea, vomiting, and altered taste exacerbate malnutrition. Reduced muscle strength hinders ambulatory ability, creating a disabling vicious cycle.

In our umbrella review, we noticed that various criteria had been employed to define sarcopenia in patients with lung cancer. The muscle mass at the third lumbar vertebra level (L3) upon CT imaging was the mostly used standard because it closely reflected the whole body fat-free mass [60]. Instead of the L3 landmark, some researchers calculated the mass at the thoracic muscle because it is related to the respiratory muscle condition [19]. Nishumura et al. [19] emphasized that the vertebral level of measurement did not interfere with the predictive value of sarcopenia for postoperative complications.

A handful of techniques can be used to determine body composition. Although DEXA and BIA are cost-effective, their estimations can be altered by the individual’s hydration status (which is often abnormal in the ill) along with the inconsistencies across different instrument brands and reference populations [11]. In contrast, CT can provide detailed imaging of specific tissues. Moreover, the examination is routinely performed throughout the cancer workup and follow-up. However, CT only measures the muscle quantity. It is unclear whether the diagnosis of sarcopenia, without assessing muscle strength, affects the predictive value. Additionally, there was considerable heterogeneity in the cutoff values, among which the L3 SMI thresholds proposed by Prado et al. [30] (men: < 52.4 cm^2^/m^2^; women < 38.5 cm^2^/m^2^) and Martin et al. [31] (men: SMI < 43 cm^2^/m^2^ for those with body mass index [BMI] < 25 kg/m^2^, SMI < 53 cm^2^/m^2^ for those with BMI ≥ 25 kg/m^2^; women: SMI < 41 cm^2^/m^2^) were the most widely adopted. Kawaguchi et al. [24] suggested that L3 PMIs < 6.36 cm^2^/m^2^ for men, < 3.92 cm^2^/m^2^ for women and < 3.70 cm^2^/m^2^ for men, < 2.50 cm^2^/m^2^ for women were optimal for predicting survival and postoperative complications, respectively. Further studies are needed to establish the most suitable cutoff values of lean body mass for the association of various prognostic parameters in patients with lung cancer.

### The impact of sarcopenia on the prognosis of lung cancer

Sarcopenia is a strong predictor of increased postoperative complications. Prior studies have delineated the deteriorating influence of sarcopenia on invasive procedures, such as hip fracture surgery, emergent abdominal surgery, and gastrectomy for cancer [61–63]. Adequate nutrition and tissue perfusion are the basis for wound healing. However, sarcopenia is associated with anemia; therefore, it impedes tissue regeneration [64]. Respiratory muscles of sarcopenic patients are weakened by hypercatabolic state and increased levels of pro-inflammatory cytokines such as interleukin (IL)-6, tumor necrosis factor (TNF)-α, and transforming growth factor (TGF)-β [65]. The ensuing difficulty of weaning from ventilator support could predispose patients to further deconditioning, pulmonary infections, longer intensive care unit stay, and ultimately death. The risk of acute respiratory failure and 30-day mortality were significantly higher in sarcopenic patients with lung cancer after pneumonectomy [39].

Current evidence suggests that patients with NSCLC and sarcopenia have inferior responsiveness to immunotherapy and progression-free survival. The goal of immunotherapy is to enhance immune surveillance, such as deploying T cells to eradicate cancer cells [66]. Muscles regulate the immune response by signaling soluble myokines, cell surface molecules, and cell-to-cell interactions [67]. Wasting of skeletal muscles is likely to disrupt the equilibrium of muscle-immune systems and impair immune cell production. Furthermore, T cells become functionally incompetent in patients with cancers due to this miscommunication between skeletal muscles and lymphoid organs [68]. The “exhausted” T cells may in turn compromise the efficacy of immunotherapy [68]. The action of immunotherapy in patients with lung cancers may also be modulated by the gut and lung microbiome (gut-lung axis) [69]. Malnutrition, chronic infections, and antibiotic overuse presumably distort the intrinsic gut ecosystem, leading to a subsequent pro-inflammatory status and sarcopenia [69].

There are inconsistent results regarding the association between sarcopenia and disease-free survival in patients with lung cancer. This may be due to the limited number of original studies conducted in the early years. In the meta-analyses by Deng et al. [18] and Yang et al. [20], disease-free survival was computed from the same three studies [33, 50, 51]. Although both reviews noted a trend towards poor disease-free survival for sarcopenic patients, neither of them revealed a statistically significant difference. Later, Kawaguchi et al. [24] demonstrated shortened disease-free survival for sarcopenic patients with surgically treated NSCLC based on six studies [33, 43, 50, 51, 70, 71]. Nevertheless, our umbrella review showed that meta-analyses on the direct impact of sarcopenia on cancer recurrence, distant metastasis, and toxicity of chemotherapy and radiotherapy were lacking.

Sarcopenia predicted poor overall survival in patients with lung cancer; similarly, sarcopenia had a negative impact on the survival of patients with operated NSCLC. Although Deng et al. [18] reported that the predictive value was more robust for stage I patients, merely one study [72] analyzing stage I–IV patients reported no significant impact of sarcopenia on the overall survival. The prognosis was also inferior in sarcopenic patients with NSCLC receiving immunotherapy. Notably, there are limited data on the survival outcomes of patients receiving chemotherapy. The mechanism by which loss of muscle mass shortens lung cancer survival can be interpreted in several ways. First, sarcopenia on its own is related to increased all-cause mortality regardless of age and sex [73]. Second, performance status, which has recently been included in the recent diagnostic criteria of the Asian Working Group for Sarcopenia, is recognized as a prognostic factor for lung cancer [74]. Deteriorated physiological reserve, a hallmark of frailty and sarcopenia, lowers the patient’s tolerance to aggressive therapeutic approaches, resulting in substandard dosing or premature treatment termination. Studies have shown that sarcopenic cancer patients had poor compliance during chemotherapy [75]. Lastly, hampered treatment response and added complication risks in sarcopenic patients, as also shown in our review, have adverse effects on cancer prognosis.

Our umbrella review has some limitations. First, it was inherently subject to biases in the included systematic reviews and meta-analyses. Complex interactions among skeletal muscles, inflammation, and the immune system are elusive; thus, there is a knowledge gap between the mechanism and treatment of sarcopenia in patients with lung cancer. Further research is needed to clarify the influence of sarcopenia on metastasis, recurrence, treatment response/toxicity, and quality of life in patients with lung cancer. Likewise, future studies verifying the predictive power of sarcopenia for various clinical outcomes in different subtypes and stages of lung cancer are also needed.

## Conclusions

Sarcopenia is a major health threat in lung cancer, affecting up to half of all patients. Its diagnosis in this population should not be underestimated because of its association with elevated postoperative complications, decreased immunotherapy response rates, and increased mortality. In patients with sarcopenia and lung cancer, survival is adversely affected regardless of the cancer type (NSCLC/SCLC), stage, or treatment option. Therefore, sarcopenia is a robust prognostic factor for therapeutic responses and outcomes in patients with lung cancer. Further research is needed regarding the pathophysiology and interventions in the lung cancer population.

## Supplementary Information

**Additional file 1: Table S1**. Keywords and search results in different databases. **Table S2**. Excluded reviews and reasons. **Table S3**. Original studies in the included reviews.

## Data Availability

Not applicable.
