# Supplementary material for: Impact of sarcopenia on the prognosis and treatment of lung cancer: an umbrella review
Source: Discov Oncol. 2022 Oct 28;13:115. doi: 10.1007/s12672-022-00576-0 (PMC9616989; doi:10.1007/s12672-022-00576-0)
Supplement: Supplementary file 1 — Additional file 1: Table S1. Keywords and search results in different databases. Table S2. Excluded reviews and reasons. Table S3. Original studies in the included reviews. [file 12672_2022_576_MOESM1_ESM.docx]

**Supplementary Table 1.** Keywords and search results in different databases

| **Database** | **Keyword** | **Filter** | **Date** | **Results** |
| --- | --- | --- | --- | --- |
| PubMed | (‘cancer’ OR ‘lung cancer’ OR ‘lung neoplasm’ OR ‘lung tumor’) AND (‘sarcopenia’ OR ‘skeletal muscle’ OR ‘muscle loss’ OR ‘nutrition’) AND (‘systematic review’ OR ‘meta-analysis’) | Title  Abstract | 2022/6/28 | 624 |
| Embase | (‘cancer’ OR ‘lung cancer’ OR ‘lung neoplasm’ OR ‘lung tumor’) AND (‘sarcopenia’ OR ‘skeletal muscle’ OR ‘muscle loss’ OR ‘nutrition’) AND (‘systematic review’ OR ‘meta-analysis’) | Title  Abstract | 2022/6/28 | 728 |
| Web of Science | (‘cancer’ OR ‘lung cancer’ OR ‘lung neoplasm’ OR ‘lung tumor’) AND (‘sarcopenia’ OR ‘skeletal muscle’ OR ‘muscle loss’ OR ‘nutrition’) AND (‘systematic review’ OR ‘meta-analysis’) | Abstract | 2022/6/28 | 424 |
| Cochrane | (‘cancer’ OR ‘lung cancer’ OR ‘lung neoplasm’ OR ‘lung tumor’) AND (‘sarcopenia’ OR ‘skeletal muscle’ OR ‘muscle loss’ OR ‘nutrition’) | Abstract | 2022/6/28 | 21 |

**Supplementary Table 2.** Excluded reviews and reasons

| **Reasons** | **References** |
| --- | --- |
| Not performing subgroup analysis on lung cancer | [1-15] |
| Reporting other nutritional measurements rather than sarcopenia | [16-19] |

**Supplementary Table 3.** Original studies in the included reviews

| **Author,**  **year** | **Study design** | **Cancer, stage** | **Number of lung cancer patients** | **Number of sarcopenic patients** | **Skeletal muscle assessment** | **Cutoff value for sarcopenia** | **Anticancer treatment** | **Main findings** |
| --- | --- | --- | --- | --- | --- | --- | --- | --- |
| **Collins et al., 2014[20]** | | | | | |  |  |  |
| Hansell et al., 1986[21] | Cross-  sectional | Lung cancer, stage unspecified | n = 12  (98 cancer patients) | NA | Titrated saline, upper arm measurements | NA | NA | Resting energy expenditure correlated with body weight and lean body mass. Resting energy expenditure was not increased in lung cancer and contributed little to cancer cachexia. |
| Fredrix et al.,  1990[22] | Cross-sectional | Lung cancer, stage unspecified | n = 17  (39 cancer patients) | NA | BIA | NA | NA ≥10 | Weight loss in lung cancer patients represented an increased resting energy expenditure and decreased energy intake. |
| Simons et al., 1997[23] | Cross- sectional | NSCLC,  stage I-IV | n = 38 | NA | DEXA | NA | NA | Elevated leptin levels were not involved in cachexia development. |
| Staal-van den Brekel et al., 1997 | Longitudinal | SCLC, limited or extended | n = 12 | NA | BIA | NA | Chemotherapy | Resting energy expenditure significantly decreased after chemotherapy. |
| Simons et al.,  1999[24] | Cross-sectional | Lung cancer, stage I-IV | n = 20 | NA | DEXA | NA | NA | Weight loss in lung cancer was associated with systemic inflammation, increased acute phase response, and decreased levels of IGF-I. |
| Jagoe et al., 2001[25] | Cross-sectional | Lung cancer, stage I-IV | n = 60  (and 22 controls) | NA | BIA, grip strength, four skinfold method | NA | Surgery | Weight, grip strength and skin fold thickness were not significantly different between patients referred for lung cancer surgery and healthy controls. |
| Jatoi et al.,  2001[26] | Case-control | NSCLC,  stage I-III | n = 18  (and 18 controls) | NA | DEXA, tritium dilution | NA | NA | Lung cancer patients manifested an increased resting energy expenditure. |
| McMillan et al., 2001[27] | Cross-sectional | NSCLC,  stage III-IV | n = 60 | NA | Total body potassium | NA | NA | Albumin concentrations were significantly correlated with reported body weight, body cell mass and CRP in advanced cancer patients. |
| Scott, et al.,  2001[28] | Longitudinal | NSCLC, locally advanced, stage unspecified | n = 60  (and 7 controls) | NA | Total body potassium | NA | NA | Resting energy expenditure was higher in the NSCLC group and correlated with magnitude of inflammatory response. |
| Agteresch et al., 2002[29] | Longitudinal RCT | NSCLC, stage IIIB-IV | n = 58 | NA | Skinfold thickness, deuterium dilution | NA | NA | ATP infusion in advanced NSCLC can inhibit weight loss and maintain appetite. |
| Crown et al.,  2002[30] | Longitudinal case-control | NSCLC, inoperable, stage unspecified | n = 30 | NA | BIA, upper arm measurements | NA | NA | Growth factor resistance, which correlated with IL-6 concentration, was observed in NSCLC patients. |
| Jagoe et al., 2002[31] | Cross-sectional | Lung cancer, stage I-IV | n = 36  (and 10 controls) | NA | BIA, four skinfold method | NA | NA | Gene expression of the lysosomal protease cathepsin B was increased in the skeletal muscle of patients with lung cancer, and negatively correlated with fat-free mass. |
| Harvie et al., 2003[32] | Longitudinal | NSCLC,  stage III-IV | n = 50 | NA | Four skinfold method | NA | NA | Fat free mass and resting energy expenditure decreased during chemotherapy in men with NSCLC. However, the trend is not seen in women with NSCLC. |
| Sarhill et al.,  2003[33] | Cross-sectional | Lung cancer, advanced, stage unspecified | n = 63  (352 cancer patients) | NA | BIA, upper arm measurements | NA | NA | Most patient referred to palliative care have severe weight loss, particularly males. Weight loss was predictive of death in advanced cancer patients. |
| Bauer et al.,  2005[34] | Longitudinal | NSCLC,  stage unspecified | n = 2  (7 cancer patients) | NA | Deuterium dilution | NA | Gemcitabine +/- other chemotherapy | There were improvements in energy intake, weight, lean body mass, performance status, quality of life with nutritional interventions given during chemotherapy. |
| Harvie et al.,  2005[35] | Longitudinal | NSCLC,  stage III-IV |  | NA | Four skinfold method | NA | Chemotherapy | Patients with NSCLC had elevated resting energy expenditure. Also, there were negative correlations between mean CRP and change in weight in NSCLC. |
| Fearon et al.,  2006[36] | RCT | Lung cancer, stage unspecified | n = 231  (518 cancer patients) | NA | BIA | NA | NA | There were no statistically significant improvements in survival, weight, or other nutritional variables with single agent EPA. However, there was a borderline favorable change in weight at eight weeks. |
| Wieland et al., 2007[37] | Longitudinal | NSCLC,  stage IIIB-IV | n = 181  (286 cancer patients) | NA | CT | NA | NA | In NSCLC patients, proteolysis-inducing factor was unrelated to survival and muscle loss. |
| Bovio et al.,  2018[38] | Cross-sectional | Lung cancer, stage unspecified | n = 144  (46 cancer patients) | NA | Upper arm measurements | NA | NA | 63% of men and 19% of women had arm muscle area below the 5th percentile. Prealbumin, transferrin and albumin levels were below normal in more than 70% of patients. |
| Prado et al.,  2008[39] | Longitudinal | NSCLC,  stage I-IV | n = 60  (250 cancer patients) | 60 | CT | Men: SMI at L3 < 52·4 cm²/m²  Women: SMI at L3 < 38·5 cm²/m² | NA | Sarcopenic obesity was independent of TNM stage and history of previous weight loss. But it was associated with poorer functional status compared with obese patients, and was an independent predictor of survival. |
| Tozer et al.,  2008[40] | RCT | Lung cancer, stage unspecified | n = 66 | NA | BIA, grip strength | NA | NA | Patients treated with cysteine-rich protein had a mean increase of 2.5% body weight, whereas casein-treated patients lost 2.6%. Secondary endpoints included an increase in survival, grip force, and quality of life. |
| Trutschnigg et al., 2008[41] | Cross-sectional | NSCLC, advanced, stage unspecified | NA  (81 cancer patients) | NA | DEXA, BIA, grip strength | NA | NA | The test-retest reliability was high for both DEXA and BIA measurements. The two could not be used interchangeably due to wide limits of agreement. |
| Beijer et al.,  2009[42] | LongitudinalRCT | Lung cancer, preterminal, stage unspecified | n = 44  (100 cancer patients) | NA | Upper arm measurements | NA | NA | There was a significant favorable effect of ATP on triceps skinfold thickness and short-term survival (0–8 weeks HR: 0.40, 95% CI: 0.17–0.95). In weight-stable patients and in lung cancer patients, long-term survival (0–6 months) was also significantly better with ATP treatment. |
| Vigano et al.,  2009[43] | Cross-sectional | NSCLC,  stage III-IV | n = 64  (172 cancer patients) | NA | DEXA, grip strength | NA | NA | Angiotensin-converting enzyme gene polymorphism was related to hemoglobin, body composition and grip strength. |
| Baracos, 2010[44] | Cross-sectional | NSCLC,  stage III-IV | n = 441 | n = 206 | CT | Men: SMI at L3 < 55.4 cm^2^/m^2^  Women: SMI at L3 < 38.9 cm^2^/m^2^ | NA | Sarcopenia was found in 61.1% of men and 31.3% of women in the cohort. Overall prevalence was 46.8%. |
| Kilgour et al.,  2010[45] | Cross-sectional | Lung cancer, stage III-IV | n = 16  (84 cancer patients) | NA | DEXAS, grip strength, quadriceps strength | NA | NA | Cancer-related fatigue was associated with grip strength, quadriceps strength and SMI. |
| Murphy et al., 2010[46] | Longitudinal | NSCLC,  stage I-IV | n = 41 | n = 25 | CT | Men: SMI at L3 < 55.4 cm^2^/m^2^  Women: SMI at L3 < 38.9 cm^2^/m^2^ | Platinum-based chemotherapy | Patients with sarcopenia had lower plasma EPA, DHA, and plasma (n-3) fatty acids. |
| Murphy et al., 2011[47] | Longitudinal | NSCLC,  stage III-IV | n = 40 | n = 18 | CT | Men: SMI at L3 <55.4 cm^2^/m^2^  Women: SMI at L3 <38.9 cm^2^/m^2^ | Platinum-based doublet chemotherapy | Patients receiving 2.2g of fish oil per day maintained their weight while the ones did not lost and average of 1kg of muscle. |
| Martinez-Hernandez et al.,  2012[48] | Longitudinal | Lung cancer, stage unspecified | n = 13  (21 cancer patients and 8 controls) | NA | BIA | NA | NA | Changes of IL-15 were directly associated with body weight changes, BMI, fat-free mass and muscle mass in cancer patients. |
| Op den Kamp et al., 2012[49] | Cross-sectional | NSCLC,  stage I-IIIB | n = 16  (and 10 controls) | NA | DEXA | NA | NA | Patients showed increased plasma levels of CRP, soluble tumor necrosis factor receptor 1, fibrinogen and decreased levels of albumin. No change in fat free body mass or upregulation of ubiquitin proteasome system was observed. |
| Peddle-Mclntyre et al., 2012[50] | Longitudinal | Lung cancer, stage I-IIIB | n = 17 | NA | DEXA | NA | NA | Muscular strength, muscle endurance and 6-minute walk test significantly improved without significant changes in body composition after progressive resistance training in lung cancer survivors. |
| Winter et al., 2012[51] | Longitudinal | NSCLC,  stage III-IV | n = 10  (and 10 controls) | NA | DEXA, grip strength | NA | NA | Serum insulin, IGF-1, C-peptide and glucagon concentrations did not differ between groups. However, NSCLC patients with moderate cachexia showed considerable insulin resistance of glucose and of whole-body protein anabolism. |
| Martin et al.,  2013, [52] | Longitudinal | Lung cancer, stage unspecified | n = 440  (1473 cancer patients) | NA | CT | Men: SMI at L3 < 43 cm^2^/m^2^ for those BMI < 25 kg/m^2^, < 53 cm^2^/m^2^ for those BMI ≥ 25 kg/m^2^,  Women: SMI at L3 < 41 cm^2^/m^2^  Men/women: SMD < 33 HU for those BMI<25kg/m^2^ and SMD< 41 for those BMI≥25kg/m^2^ | NA | High weight loss, low muscle index, and low muscle attenuation were independently prognostic of survival. |
| Op den Kamp et al., 2013[53] | Cross-sectional | NSCLC,  stage IIIB-IV | n = 26  (and 22 controls) | NA | DEXA | NA | NA | Appendicular lean mass atrophy was evident in cachectic patients compared to healthy controls. mRNA transcript expression of the negative muscle mass regulator myostatin and protein phosphorylation of its downstream signaling constituents was not altered in lung cancer. |
| Prado et al.,  2013[54] | Longitudinal | NSCLC,  stage IIIB-IV | n = 242  (368 cancer patients) | NA | CT | NA | NA | Being within 90 days from death was a major risk factor for muscle loss (OR: 2.67, 95%CI: 1.45-4.94). Patient become more catabolic approaching death. |
| **Buentzel et al., 2019[55]** | | | | | |  |  |  |
| Gupta et al., 2009[56] | Retrospective cohort | NSCLC, stage IIIB-IV | n = 165 | NA | BIA | Phase angle ≤ 5.3 degrees | NA | Phase angle, tumor stage and treatment history were statistically significantly associated with survival. Every one degree increase in phase angle was associated with a relative risk of 0.79. |
| Sanchez-Lara et al., 2012[57] | Longitudinal | NSCLC,  stage IIIB-IV | n = 119 |  | BIA | Phase angle ≤ 5.8 degrees |  | ECOG 2 (HR: 2.7, 95%CI: 1.5–4.7), phase angle ≤5.8 degrees (HR: 3.02, 95%CI: 1.2–7.11) were associated with poor survival. |
| Kim et al.,  2015[58] | Retrospective cohort | SCLC, limited or extended | n = 149 | n = 118 | CT | Men: SMI at L3 < 55 cm^2^/m^2^  Women: SMI at L3 < 39 cm^2^/m^2^ | Chemotherapy, radiotherapy, palliative care | Prevalence of sarcopenia was 79.2% and was significantly related to an advanced age, male sex, lower BMI, and poor performance status. Sarcopenic patients had shorter overall survival. |
| Kimura et al.,  2015[59] | Retrospective cohort | NSCLC,  stage IIIA-IV | n = 134 | n = 36 | CT | Men: SMI at L3 < 41 cm^2^/m^2^  Women: SMI at L3 < 38 cm^2^/m^2^ | Chemotherapy, target therapy | Cachexia presence and decreased skeletal muscle mass are associated with poor prognosis in advanced NSCLC. |
| Stene et al., 2015[60] | Longitudinal | NSCLC,  stage IIIB-IV | n =35 | n = 26 | CT | Men: SMI at L3 < 52·4 cm²/m²  Women: SMI at L3 < 38·5 cm²/m² | Palliative chemotherapy | Maintaining or gaining muscle resulted in longer median overall survival. Muscle loss but not sarcopenia at baseline, was a significant prognostic factor. |
| Sjoblom et al., 2016[61] | Longitudinal | NSCLC,  stage IIIB-IV | n = 734 | n = 213 by SMI  n = 74 by SMD | CT | Men: SMI at L3< 47.6 cm²/m², SMD < 28 HU  Women: SMD < 23.8 HU | Chemotherapy | SMD was independently prognostic for survival, whereas SMI was not. |
| Suzuki et al.,  2016[62] | Retrospective  cohort | NSCLC,  stage IA-IB | n = 90 | n = 38 | CT | Men: SMI at L3 < 43.75 cm^2^/m^2^  Women: SMI at L3 < 41.10 cm^2^/m^2^ | Surgery | Patients with sarcopenia were more likely to have a low BMI. Multivariate analysis found that sarcopenia was a significant independent prognostic factor (HR: 7.09). |
| Bowden et al., 2017[63]  2017[63] | Retrospective cohort | NSCLC, stage II-IV  SCLC, limited or extended | n = 194 | n = 53 by SMI  n= 72 by SMD | CT | Men: SMI at T4 < 67.3 cm²/m², SMD < 44.1 HU  Women: SMI at T4 < 46.3 cm²/m², SMD < 40.5 HU | Chemotherapy, radiotherapy | BMI < 20 and ECOG performance status 2 were independent predictors of death within 90 days of treatment completion. Patients with low muscle attenuation had significantly reduced overall survival. |
| Kinsey et al., 2017[64] | Longitudinal | NSCLC,  stage I-IV | n = 252 | n = 126 | CT | Men: pectoralis muscle area < 50th percentile in the cohort  Women: pectoralis muscle area < 50th percentile in the cohort | Surgery, chemotherapy, radiotherapy | Lower pectoralis muscle area at diagnosis was associated with worse overall survival for NSCLC patients regardless of sex, stage, smoking, chronic bronchitis, BMI and performance status. |
| Shoji et al., 2017[65] | Retrospective  cohort | NSCLC,  stage IA-IIA | n = 147 | n = 57 | CT | Men: SMI at L3 < 43.75 cm^2^/m^2^  Women: SMI at L3 < 41.10 cm^2^/m^2^ | Surgery +/- adjuvant chemotherapy | Pre-operative sarcopenia was significantly associated with poor survival (HR: 5.138, 95%CI: 2.305-11.676) and immune-nutritional parameters in early NSCLC. |
| Tsukioka et al., 2017[66] | Retrospective cohort | NSCLC,  stage I | n = 215 | n =30 | CT | Men: SMI at L3 < 49 cm²/m²  Only male patients were included | Surgery | Sarcopenia was significantly associated with BMI, nutritional condition, serum CYFRA 21-1 level and pathological stage, but not with pre-operative respiratory function or performance status. The sarcopenia group had a significantly shorter median overall survival than the non-sarcopenia group. |
| Chambard et al., 2018[67] | Longitudinal | Lung adenocarcinoma with bone metastasis, stage IV | n = 64 | n = 16 | DEXA | Men: ALM < 7.26 kg/m2 for Women: ALM < 5.45 kg/m2 | Chemotherapy, target therapy, antiresorptive treatment, palliative care | Sarcopenia was associated with poorer survival (HR: 2.96, 95%CI:1.40–6.27). |
| Mitsuyoshi et al., 2018[68] | Retrospective cohort | NSCLC,  stage IIIA-IIIB | n = 89 | n = 22 | CT | Men: PMI at L3 < 3.73 cm^2^/m^2^  Women: PMI at L3 < 2.45 cm^2^/m^2^ | Chemotherapy, radiotherapy | Elevated CRP (≥0.3 mg/dl) and low BMI (<18.5 kg/m2) were correlated with shorter overall survival, but albumin level and SMI were not. |
| Rossi et al., 2018[69] | Retrospective cohort | NSCLC,  stage IV | n = 33 | n = 20 | CT | Men: SMI at L3 ≤ 55 cm^2^/m^2^  Women: SMI at L3 ≤ 39 cm^2^/m^2^ | Gefitinib | Sarcopenia did not affect response to gefitinib treatment in EGFR mutated NSCLC patients, even if it is a bad prognostic indicator for overall survival. |
| Takamori et al., 2018[70] | Retrospective cohort | NSCLC,  stage I | n = 101 | NA | CT | 10% loss of SMA at T12 | Surgery +/- adjuvant chemotherapy | Decreased SMA was an independent prognostic factor for disease free survival and overall survival. Independent risk factors for skeletal muscle loss included poor performance status and obstructive ventilatory impairment. |
| **Deng et al., 2019[71]** | | | | |  |  |  |  |
| Suzuki et al.,  2016[62] | Retrospective  cohort | NSCLC,  stage IA-IB | n = 90 | n = 38 | CT | Men: SMI at L3 < 43.75 cm^2^/m^2^  Women: SMI at L3 < 41.10cm^2^/m^2^ | Surgery | Patients with sarcopenia were more likely to have a low BMI. Multivariate analysis found that sarcopenia was a significant independent prognostic factor (HR: 7.09). |
| Hervochon et al., 2017[72] | Retrospective cohort | NSCLC,  stage I-IV | n = 161 | n =53 | CT | Men: PMA at L3 ≤ 1601 mm^2^  Women: PMA at L3 ≤ 999 mm^2^ | Surgery | Sarcopenia was associated with high CRP levels (>20 mg/L). Multivariate analysis showed the independent prognostic value of both BMI and total psoas area. |
| Shoji et al., 2017[65] | Retrospective  cohort | NSCLC,  stage IA-IIA | n = 147 | n = 57 | CT | Men: SMI at L3 < 43.75 cm^2^/m^2^  Women: SMI at L3 < 41.10cm^2^/m^2^ | Surgery +/- adjuvant chemotherapy | Pre-operative sarcopenia was significantly associated with poor survival (HR: 5.138, 95%CI: 2.305-11.676) and immune-nutritional parameters in early NSCLC. |
| Tsukioka et al., 2017[66] | Retrospective cohort | NSCLC,  Stage I | n = 215 | n =30 | CT | Men: SMI at L3 < 49 cm²/m²  Only male patients were included | Surgery | Sarcopenia was significantly associated with BMI, nutritional condition, serum CYFRA 21-1 level and pathological stage, but not with pre-operative respiratory function or performance status. The sarcopenia group had a significantly shorter median overall survival than the non-sarcopenia group. |
| Kim et al., 2017[73] | Retrospective cohort | NSCLC,  stage I-IV | n = 272 | n = 61 | CT | Men: SMI at L3 < 55 cm^2^/m^2^  Women: SMI at L3 < 39 cm^2^/m^2^ | Surgery | The prevalence of sarcopenia was 22.4%. No significant difference was observed between patients with or without sarcopenia in terms of ICU or hospital stay, post-operative complications, 3-year disease free survival or 3-year overall survival. |
| Nakamura et al., 2018[74] | Retrospective cohort | NSCLC,  stage I-III | n = 328 | n = 183 | CT | Men: PMI at L3 < 6.36 cm^2^/m^2^  Women: PMI at L3 < 3.92 cm^2^/m^2^ | Surgery | Sarcopenia was significantly related with increasing age, male sex, smoking habit, lower BMI, and post-operative major complication. Multivariate analysis found that sarcopenia was an independent unfavorable prognostic factor for survival. |
| **Nishimura et al., 2019[75]** | | | | | | |  |  |
| Suzuki et al.,  2016[62] | Retrospective  cohort | NSCLC,  stage IA-IB | n = 90 | n = 38 | CT | Men: SMI at L3 < 43.75 cm^2^/m^2^  Women: SMI at L3 < 41.10cm^2^/m^2^ | Surgery | Patients with sarcopenia were more likely to have a low BMI. Multivariate analysis found that sarcopenia was a significant independent prognostic factor (HR: 7.09). |
| Hervochon et al., 2017[72] | Retrospective cohort | NSCLC,  stage I-IV | n = 161 | n =53 | CT | Men: PMA at L3 ≤ 1601 mm^2^  Women: PMA at L3 ≤ 999 mm^2^ | Surgery | Sarcopenia was associated with high CRP levels (>20 mg/L). Multivariate analysis showed the independent prognostic value of both BMI and total psoas area. |
| Shoji et al., 2017[65] | Retrospective  cohort | NSCLC,  stage IA-IIA | n = 147 | n = 57 | CT | Men: SMI at L3 < 43.75 cm^2^/m^2^  Women: SMI at L3 < 41.10 cm^2^/m^2^ | Surgery +/- adjuvant chemotherapy | Pre-operative sarcopenia was significantly associated with poor survival (HR: 5.138, 95%CI: 2.305-11.676) and immune-nutritional parameters in early NSCLC. |
| Fintelmann et al., 2018[76] | Retrospective cohort | NSCLC,  stage I-IV | n = 135 | n = 67 | CT | Men: SMA at T5 < 181.2 cm^2^  Women: SMA at T5 < 129.4 cm^2^ | Surgery | Low thoracic muscle was independently associated with increased post-operative complications and health care utilization among patients undergoing lobectomy for lung cancer. |
| Kim et al., 2017[73] | Retrospective cohort | NSCLC,  stage I-IV | n = 272 | n = 61 | CT | Men: SMI at L3 < 55 cm^2^/m^2^  Women: SMI at L3 < 39 cm^2^/m^2^ | Surgery | The prevalence of sarcopenia was 22.4%. No significant difference was observed between patients with or without sarcopenia in terms of ICU or hospital stay, post-operative complications, 3-year disease free survival rate or survival rate. |
| Miller et al., 2018[77] | Retrospective cohort | Lung cancer and tumors metastatic to the lung, stage unspecified | n = 299 | NA | CT | SMI at T12 analyzed as a continuous variable | Surgery | The height adjusted-erector spinae muscle cross sectional area was significantly associated with 30-day mortality and hospital length of stay. |
| Nakamura et al., 2018[74] | Retrospective cohort | NSCLC,  stage I-III | n = 328 | n = 183 | CT | Men: PMI at L3 < 6.36 cm^2^/m^2^  Women: PMI at L3 < 3.92 cm^2^/m^2^ | Surgery | Sarcopenia was significantly related with increasing age, male sex, smoking habit, lower BMI, and post-operative major complication. Multivariate analysis found sarcopenia independently predicted poor survival. |
| Takamori et al., 2018[70] | Retrospective cohort | NSCLC,  stage I | n = 101 | NA | CT | 10% loss of SMA at T12 | Surgery +/- adjuvant chemotherapy | Decreased SMA was an independent prognostic factor for worse disease free survival and overall survival. Independent risk factors for skeletal muscle loss included poor performance status and obstructive ventilatory impairment. |
| Troschel et al., 2019[78] | Retrospective cohort | Lung cancer, stage I-IV | n = 128 | n =64 | CT | Men: SMA at T8 < 115.3 cm2  Women: SMA at T8 < 74.0 cm2 | Surgery | Multivariable analysis showed an independent association of muscle CSA and overall survival with a hazard ratio of 0.80 (95%CI: 0.67–0.98). |
| **Yang et al., 2019[79]** | | | |  |  |  |  |  |
| Kim et al.,  2015[58] | Retrospective cohort | SCLC, limited or extended | n = 149 | n = 118 | CT | Men: SMI at L3 < 55 cm^2^/m^2^  Women: SMI at L3 < 39 cm^2^/m^2^ | Chemotherapy, radiotherapy, palliative care | Prevalence of sarcopenia was 79.2% and was significantly related to an advanced age, male sex, lower BMI, and poor performance status. Sarcopenic patients had shorter overall survival. |
| Kimura et al.,  2015[59] | Retrospective cohort | NSCLC,  stage IIIA-IV | n = 134 | n = 36 | CT | Men: SMI at L3 < 41 cm^2^/m^2^  Women: SMI at L3 < 38 cm^2^/m^2^ | Chemotherapy, target therapy | Cachexia presence and decreased skeletal muscle mass are associated with poor prognosis in advanced NSCLC. |
| Stene et al., 2015[60] | Longitudinal | NSCLC,  stage IIIB-IV | n =35 | n = 26 | CT | Men: SMI at L3 < 52·4 cm²/m²  Women: SMI at L3 < 38·5 cm²/m² | Palliative chemotherapy | Maintaining or gaining muscle resulted in longer median overall survival. Muscle loss but not sarcopenia at baseline, was a significant prognostic factor. |
| Go et al, 2016[80] | Retrospective cohort | SCLC, limited or extended | n = 117 | n = 29 | CT | Men: SMI at T4 < 437 mm^2^ /m^2^  Only male patients were included | Chemotherapy, radiotherapy | Sarcopenic patients had lower progression free survival than did non-sarcopenic patients, but the difference in overall survival was not significant. Early discontinuation of treatment and treatment-related mortality happened more in the sarcopenic group. |
| Srdic et al., 2016[81] | Longitudinal | NSCLC,  stage IIIB-IV | n = 55 | n = 30 | CT | Men: SMI at L3 < 55 cm^2^/m^2^  Women: SMI at L3 < 39 cm^2^/m^2^ | Chemotherapy | Cachexia and sarcopenia were not found to be predictors of chemotoxicity nor was time to tumor progression. |
| Suzuki et al.,  2016[62] | Retrospective  cohort | NSCLC,  stage IA-IB | n = 90 | n = 38 | CT | Men: SMI at L3 < 43.75 cm^2^/m^2^  Women: SMI at L3 < 41.10cm^2^/m^2^ | Surgery | Patients with sarcopenia were more likely to have a low BMI. Multivariate analysis found that sarcopenia was a significant independent prognostic factor (HR: 7.09). |
| Shoji et al., 2017[65] | Retrospective  cohort | NSCLC,  stage IA-IIA | n = 147 | n = 57 | CT | Men: SMI at L3 < 43.75 cm^2^/m^2^  Women: SMI at L3 < 41.10 cm^2^/m^2^ | Surgery +/- adjuvant chemotherapy | Pre-operative sarcopenia was significantly associated with poor survival (HR: 5.138, 95%CI: 2.305-11.676) and immune-nutritional parameters in early NSCLC. |
| Tsukioka et al., 2017[66] | Retrospective cohort | NSCLC,  Stage I | n = 215 | n =30 | CT | Men: SMI at L3 < 49 cm²/m²  Only male patients were included | Surgery | Sarcopenia was significantly associated with BMI, nutritional condition, serum CYFRA 21-1 level and pathological stage, but not with pre-operative respiratory function or performance status. The sarcopenia group had a significantly shorter median overall survival than the non-sarcopenia group. |
| Chambard et al., 2018[67] | Longitudinal | Adenocarcinoma with bone metastasis, stage IV | n = 64 | n = 16 | DEXA | Men: ALM < 7.26 kg/m^2^  Women: ALM < 5.45 kg/m^2^ | Chemotherapy, target therapy, antiresorptive treatment, palliative care | Sarcopenia was associated with poorer survival (HR: 2.96, 95%CI:1.40–6.27) in lung adenocarcinoma patients with bone metastases. |
| Kim et al., 2017[73] | Retrospective cohort | NSCLC,  stage I-IV | n = 272 | n = 61 | CT | Men: SMI at L3 < 55 cm^2^/m^2^  Women: SMI at L3 < 39 cm^2^/m^2^ | Surgery | The prevalence of sarcopenia was 22.4%. No significant difference was observed between patients with or without sarcopenia in terms of ICU or hospital stay, post-operative complications, 3-year disease free survival or 3-year overall survival. |
| Matsuo et al., 2018[82] | Retrospective cohort | NSCLC,  stage IA-IIA | n = 186 | n = 94 | CT | Men: one side PMI at L3 < 293 mm^2^/m^2^  Women: one side PMI at L3 < 240 mm^2^/m^2^ | Radiotherapy | Overall survival in patients in low PMI was worse than those with high PMI. Non-lung cancer death, but not lung cancer related death, was significantly worse in the low PMI group. |
| Nakamura et al., 2018[74] | Retrospective cohort | NSCLC,  stage I-III | n = 328 | n = 183 | CT | Men: PMI at L3 < 6.36 cm^2^/m^2^  Women: PMI at L3 < 3.92 cm^2^/m^2^ | Surgery | Sarcopenia was significantly related with increasing age, male sex, smoking habit, lower BMI, and post-operative major complication. Multivariate analysis found that sarcopenia was an independent unfavorable prognostic factor for survival. |
| Rossi et al., 2018[69] | Retrospective cohort | NSCLC,  stage IV | n = 33 | n = 20 | CT | Men: SMI at L3 ≤ 55 cm^2^/m^2^  Women: SMI at L3 ≤ 39 cm^2^/m^2^ | Gefitinib | Sarcopenia did not affect response to gefitinib treatment in EGFR mutated NSCLC patients, even if it is a bad prognostic indicator for overall survival. |
| **Wang et al., 2020[83]** | | | | | |  |  |  |
| Shachar et al., 2018[84] |  | NSCLC,  stage IV | n = 78 | n = 52 | CT | men: SMI at L3 < 43 cm^2^/m^2^ for those BMI < 25 kg/m^2^, < 53 cm^2^/m^2^ for those BMI ≥ 25kg/m^2^  women: SMI at L3 < 41 cm^2^/m^2^ f | Nivolumab | Grade 3-4 toxicities happened more frequently in sarcopenic patients compared to non-sarcopenic patients. Durasion of response is shorter in non-sarcopenic patients. |
| Cortellini et al., 2019[85] | Retrospective cohort | NSCLC,  stage IV | n = 23 | n = 9 | CT | Men: SMI at L3 < 43 cm^2^/m^2^ for those BMI < 25 kg/m^2^, < 53 cm^2^/m^2^ for those BMI ≥ 25kg/m^2^  Women: SMI at L3 < 41 cm^2^/m^2^ | Nivolumab | There were trends towards longer median progression free survival and median overall survival in favor of patients without sarcopenia. However, no statistically significant differences were observed. |
| Magri et al, 2019[86] | Retrospective cohort | NSCLC, pretreated, stage unspecified | n = 46 | NA | CT | SMI at L3 analyzed as a continuous variable | Nivolumab | CT-derived parameters were found to correlate partly with BMI, but not with albumin, nor with weight loss. Weight loss during therapy was an independent poor prognostic factor. |
| Nishioka et al., 2019[87] | Retrospective cohort | NSCLC,  stage III-IV or recurrent | n = 38 | n - 21 | CT | ≥ 10% loss of PMA at L2-3 between diagnosis and starting immunotherapy | Anti-PD-1 inhibitors | Patients with sarcopenia exhibited a significantly shorter median progression free survival than non-sarcopenia patients. |
| Shiroyama et al., 2019[88] | Retrospective cohort | NSCLC, pretreated, stage unspecified | n = 42 | n = 22 | CT | Men: PMI at L3 < 6.36 cm^2^/m^2^  Women: PMI at L3 < 3.92 cm^2^/m^2^ | Anti-PD-1 inhibitors | The prevalence of sarcopenia was 52.4%. Sarcopenia was significantly associated with poorer response rate and progression free survival. |
| Minami et al., 2020[89] | Retrospective cohort | NSCLC, pretreated, stage unspecified | n = 74 | n = 53 | CT | Men: PMI at L3 < 6.36 cm^2^/m^2^  Women: PMI at L3 < 3.92 cm^2^/m^2^ | Anti-PD-(L)1 inhibitors | There was no significant difference in overall survival and progression free survival according to PMI, intramuscular adipose tissue content or visceral-to-subcutaneous ratio. |
| Roch et al.,  2020[90] | Retrospective cohort | NSCLC,  stage IV or recurrent | n = 142 | n = 92 | CT | Men: SMI at L3 < 52·4 cm²/m²  Women: SMI at L3 < 38·5 cm²/m² | Anti-PD-(L)1 inhibitors | Patients with evolving sarcopenia had a shorter progression free survival and overall survival. |
| Takada et al., 2020[91] | Retrospective cohort | NSCLC,  stage IIIB-IV or recurrent | n = 103 | n = 51 | CT | Men: SMI at L3 < 25.63 cm^2^/m^2^  Women: SMI at L3 < 21.73cm^2^/m^2^ | Anti-PD-1 inhibitors | Low SMI at L3 was an independent predictor of both progression free and overall survival. Disease control rate was also significantly lower in the sarcopenic group. No difference in response rate was seen between groups. |
| Tsukagoshi et al., 2020[92] | Retrospective cohort | NSCLC,  stage III-IV or recurrent | n = 30 | n = 13 | CT | Men: PMI at L3 < 6.36 cm^2^/m^2^  Women: PMI at L3 < 3.92 cm^2^/m^2^ | Nivolumab | There was no significant association between sarcopenia and immune-related adverse events. Muscle loss was significantly associated with fewer nivolumab cycles, poorer response rate, shorter progression-free survival, and median overall survival. |
| **Au et al., 2021[93]** | | | | | |  |  |  |
| Kim et al.,  2015[58] | Retrospective cohort | SCLC, limited or extended | n = 149 | n = 118 | CT | Men: SMI at L3 < 55 cm^2^/m^2^  Women: SMI at L3 < 39 cm^2^/m^2^ | Chemotherapy, radiotherapy, palliative care | Prevalence of sarcopenia was 79.2% and was significantly related to an advanced age, male sex, lower BMI, and poor performance status. Sarcopenic patients had shorter overall survival. |
| Kimura et al.,  2015[59] | Retrospective cohort | NSCLC,  stage IIIA-IV | n = 134 | n = 36 | CT | Men: SMI at L3 < 41 cm^2^/m^2^  Women: SMI at L3 < 38 cm^2^/m^2^ | Chemotherapy, target therapy | Cachexia presence and decreased skeletal muscle mass are associated with poor prognosis in advanced NSCLC. |
| Hervochon et al., 2017[72] | Retrospective cohort | NSCLC,  stage I-IV | n = 161 | n =53 | CT | Men: PMA at L3 ≤ 1601 mm^2^  Women: PMA at L3 ≤ 999 mm^2^ | Surgery | Sarcopenia was associated with high CRP levels (>20 mg/L). Multivariate analysis showed the independent prognostic value of both BMI and total psoas area. |
| Shoji et al., 2017[65] | Retrospective  cohort | NSCLC,  stage IA-IIA | n = 147 | n = 57 | CT | Men: SMI at L3 < 43.75 cm^2^/m^2^  Women: SMI at L3 < 41.10cm^2^/m^2^ | Surgery +/- adjuvant chemotherapy | Pre-operative sarcopenia was significantly associated with poor survival (HR: 5.138, 95%CI: 2.305-11.676) and immune-nutritional parameters in early NSCLC. |
| Tsukioka et al., 2017[66] | Retrospective cohort | NSCLC,  stage I | n = 215 | n =30 | CT | Men: SMI at L3 < 49 cm²/m²  Only male patients were included | Surgery | Sarcopenia was significantly associated with BMI, nutritional condition, serum CYFRA 21-1 level and pathological stage, but not with pre-operative respiratory function or performance status. The sarcopenia group had a significantly shorter median overall survival than the non-sarcopenia group. |
| **Deng et al, 2021[94]** | | | | |  |  |  |  |
| Cortellini et al., 2019[85] | Retrospective cohort | NSCLC,  stage IV | n = 23 | n - 9 | CT | Men: SMI at L3 < 43 cm^2^/m^2^ for those BMI < 25kg/m^2^, < 53 cm^2^/m^2^ for those BMI ≥ 25 kg/m^2^  Women: SMI at L3 < 41 cm^2^/m^2^ | Nivolumab | There were trends towards longer median progression free survival and median overall survival in favor of patients without sarcopenia. However, no statistically significant differences were observed. |
| Nishioka et al., 2019[87] | Retrospective cohort | NSCLC,  stage III-IV or recurrent | n = 38 | n - 21 | CT | ≥ 10% loss of PMA at L2-3 between diagnosis and starting immunotherapy | Anti-PD-1 inhibitors | Patients with sarcopenia exhibited a significantly shorter median progression free survival than non-sarcopenia patients. |
| Shiroyama et al., 2019[88] | Retrospective cohort | NSCLC, pretreated, stage unspecified | n = 42 | n = 22 | CT | Men: PMI at L3 < 6.36 cm^2^/m^2^  Women: PMI at L3 < 3.92 cm^2^/m^2^ | Anti-PD-1 inhibitors | The prevalence of sarcopenia was 52.4%. Sarcopenia was significantly associated with poorer response rate and progression free survival. |
| Takada et al., 2020[91] | Retrospective cohort | NSCLC,  stage IIIB-IV or recurrent | n = 103 | n = 51 | CT | Men: SMI at L3 < 25.63 cm^2^/m^2^  Women: SMI at L3 < 21.73cm^2^/m^2^ | Anti-PD-1 inhibitors | Low SMI at L3 was an independent predictor of both progression free and overall survival. Disease control rate was also significantly lower in the sarcopenic group. No difference in response rate was seen between groups. |
| Tsukagoshi et al., 2020[92] | Retrospective cohort | NSCLC,  stage III-IV or recurrent | n = 30 | n = 13 | CT | Men: PMI at L3 < 6.36 cm^2^/m^2^  Women: PMI at L3 < 3.92 cm^2^/m^2^ | Nivolumab | There was no significant association between sarcopenia and immune-related adverse events. Muscle loss was significantly associated with fewer nivolumab cycles, poorer response rate, shorter progression-free survival, and median overall survival. |
| **Kawaguchi et al., 2021[95]** | | | | | | |  |  |
| Suzuki et al.,  2016[62] | Retrospective  cohort | NSCLC,  stage IA-IB | n = 90 | n = 38 | CT | Men: SMI at L3 < 43.75 cm^2^/m^2^  Women: SMI at L3 < 41.10cm^2^/m^2^ | Surgery | Patients with sarcopenia were more likely to have a low BMI. Multivariate analysis found that sarcopenia was a significant independent prognostic factor (HR: 7.09). |
| Tsukioka et al., 2017[66] | Retrospective cohort | NSCLC,  stage I | n = 215 | n =30 | CT | Men: SMI at L3 < 49 cm²/m²  Only male patients were included | Surgery | Sarcopenia was significantly associated with BMI, nutritional condition, serum CYFRA 21-1 level and pathological stage, but not with pre-operative respiratory function or performance status. The sarcopenia group had a significantly shorter median overall survival than the non-sarcopenia group. |
| Shoji et al., 2017[65] | Retrospective  cohort | NSCLC,  stage IA-IIA | n = 147 | n = 57 | CT | Men: SMI at L3 < 43.75 cm^2^/m^2^  Women: SMI at L3 < 41.10 cm^2^/m^2^ | Surgery +/- adjuvant chemotherapy | Pre-operative sarcopenia was significantly associated with poor survival (HR: 5.138, 95%CI: 2.305-11.676) and immune-nutritional parameters in early NSCLC. |
| Kim et al., 2017[73] | Retrospective cohort | NSCLC,  stage I-IV | n = 272 | n = 61 | CT | Men: SMI at L3 < 55 cm^2^/m^2^  Women: SMI at L3 < 39 cm^2^/m^2^ | Surgery | The prevalence of sarcopenia was 22.4%. No significant difference was observed between patients with or without sarcopenia in terms of ICU or hospital stay, post-operative complications, 3-year disease free survival or 3-year overall survival. |
| Nakamura et al., 2018[74] | Retrospective cohort | NSCLC,  stage I-III | n = 328 | n = 183 | CT | Men: PMI at L3 < 6.36 cm^2^/m^2^  Women: PMI at L3 < 3.92 cm^2^/m^2^ | Surgery | Sarcopenia was significantly related with increasing age, male sex, smoking habit, lower BMI, and post-operative major complication. Multivariate analysis found that sarcopenia was an independent unfavorable prognostic factor for survival. |
| Tsukioka et al., 2018[96] | Retrospective cohort | NSCLC,  stage IIIA | n = 69 | n = 21 | CT | Men: SMI at L3 < 52·4 cm²/m²  Women: SMI at L3 < 38·5 cm²/m² | Surgery | Sarcopenia was an independent predictor of poor prognosis in patients with stage IIIA NSCLC. Sarcopenia was significantly correlated with neutrophil/lymphocyte ratio elevation. |
| Kawaguchi et al., 2019[97] | Retrospective cohort | NSCLC,  stage 0, I-III | n = 173 | n = 32 | CT | Men: PMI at L3 < 3.70 cm^2^/m^2^  Women: PMI at L3 < 2.50 cm^2^/m^2^ | Surgery | Sarcopenia was a strong and independent predictor for survival in elderly NSCLC patients. |
| Icard et al., 2020[98] | Retrospective cohort | NSCLC,  stage I-IV | n =304 | n = 101 | Index derived from anthropometric measurements | Men: height indexed total muscular mass ≤ 6.49 kg/m^2^  Women: height indexed total muscular mass ≤ 6.49 kg/m^2^ | Surgery | Long-term outcome was negatively influenced by age, low muscle mass and weight loss, extent of resection, pleural invasion, and higher pathologic stage. |
| Ozeki et al.,  2020[99] | Retrospective cohort | NSCLC,  stage 0, I-III | n = 721 | n = 116 | CT | For adenocarcinoma  Men: PMI at L3 < 5.52 mm^2^/m^2^  Women: PMI at L3 < 4.07 cm^2^/m^2^  For squamous cell carcinoma  Men: PMI at L3 < 4.66 mm^2^/m^2^  Women: PMI at L3 < 3.29 cm^2^/m^2^ | Surgery | PMI was significantly associated with the survival of lung squamous cell carcinoma patients, but not of lung adenocarcinoma patients. |
| Shinohara et al., 2020[100] | Retrospective cohort | NSCLC,  stage I-IV | n =391 | n =198 | CT | Men: PMI at L3 < 6.36 cm^2^/m^2^  Women: PMI at L3 < 3.92 cm^2^/m^2^ | Surgery | Sarcopenia was an independent unfavorable prognostic factor associated with overall survival and recurrence free survival. |
| **Lee et al., 2021[101]** | | | | | |  |  |  |
| Magri et al, 2019[86] | Retrospective cohort | NSCLC, pretreated, stage unspecified | n = 46 | NA | CT | SMI at L3 analyzed as a continuous variable | Nivolumab | CT-derived parameters were found to correlate partly with BMI, but not with albumin, nor with weight loss. Weight loss during therapy was an independent poor prognostic factor. |
| Shiroyama et al., 2019[88] | Retrospective cohort | NSCLC, pretreated, stage unspecified | n = 42 | n = 22 | CT | Men: PMI at L3 < 6.36 cm^2^/m^2^  Women: PMI at L3 < 3.92 cm^2^/m^2^ | Anti-PD-1 inhibitors | The prevalence of sarcopenia was 52.4%. Sarcopenia was significantly associated with poorer response rate and progression free survival. |
| Minami et al., 2020[89] | Retrospective cohort | NSCLC, pretreated, stage unspecified | n = 74 | n = 53 | CT | Men: PMI at L3 < 6.36 cm^2^/m^2^  Women: PMI at L3 < 3.92 cm^2^/m^2^ | Anti-PD-(L)1 inhibitors | There was no significant difference in overall survival and progression free survival according to PMI, intramuscular adipose tissue content or visceral-to-subcutaneous ratio. |
| Roch et al.,  2020[90] | Retrospective cohort | NSCLC,  stage IV or recurrent | n = 142 | n = 92 | CT | Men: SMI at L3 < 52·4 cm²/m²  Women: SMI at L3 < 38·5 cm²/m² | Anti-PD-(L)1 inhibitors | Patients with evolving sarcopenia had a shorter progression free survival and overall survival. |
| Takada et al., 2020[91] | Retrospective cohort | NSCLC,  stage IIIB-IV or recurrent | n = 103 | n = 51 | CT | Men: SMI at L3 < 25.63 cm^2^/m^2^  Women: SMI at L3 < 21.73cm^2^/m^2^ | Anti-PD-1 inhibitors | Low SMI at L3 was an independent predictor of both progression free and overall survival. Disease control rate was also significantly lower in the sarcopenic group. No difference in response rate was seen between groups. |
| Tsukagoshi et al., 2020[92] | Retrospective cohort | NSCLC,  stage III-IV or recurrent | n = 30 | n = 13 | CT | Men: PMI at L3 < 6.36 cm^2^/m^2^  Women: PMI at L3 < 3.92 cm^2^/m^2^ | Nivolumab | There was no significant association between sarcopenia and immune-related adverse events. Muscle loss was significantly associated with fewer nivolumab cycles, poorer response rate, shorter progression-free survival, and median overall survival. |
| **McGovern et al., 2021[102]** | | | | |  |  |  |  |
| Stene et al., 2015[60] | Longitudinal | NSCLC,  stage IIIB-IV | n =35 | n = 26 | CT | Men: SMI at L3 < 52·4 cm²/m²  Women: SMI at L3 < 38·5 cm²/m² | Palliative chemotherapy | Prevalence of sarcopenia was 74.3%. Maintaining or gaining muscle resulted in longer median overall survival. Muscle loss but not sarcopenia at baseline, was a significant prognostic factor. |
| Suzuki et al.,  2016[62] | Retrospective  cohort | NSCLC,  stage IA-IB | n = 90 | n = 38 | CT | Men: SMI at L3 < 43.75 cm^2^/m^2^  Women: SMI at L3 < 41.10 cm^2^/m^2^ | Surgery | Prevalence of sarcopenia was 42.2%. Patients with sarcopenia were more likely to have a low BMI. Multivariate analysis found that sarcopenia was a significant independent prognostic factor (HR: 7.09). |
| Kim et al., 2016[103] | Retrospective cohort | SCLC, limited or extended | n = 186 | n = 128 | CT | Men: SMI at L3 < 55 cm2/m2  Women: SMI at L3 < 39 cm2/m2 | NA | Prevalence of sarcopenia was 68.8%. Sarcopenic patients had significantly higher neutrophil-lymphocyte ratios and CRP levels than non-sarcopenic patients. CRP independently predicted SMI and performance status. |
| Sjoblom et al., 2016[61] | Longitudinal | NSCLC,  stage IIIB-IV | n = 734 | n = 213 by SMI  n = 74 by SMD | CT | Men: SMI at L3< 47.6 cm²/m², SMD < 28 HU  Women: SMD < 23.8 HU | Chemotherapy | SMD was independently prognostic for survival, whereas SMI was not. |
| Srdic et al., 2016[81] | Longitudinal | NSCLC,  stage IIIB-IV | n = 55 | n = 30 | CT | Men: SMI at L3 < 55 cm^2^/m^2^  Women: SMI at L3 < 39 cm^2^/m^2^ | Chemotherapy | Cachexia and sarcopenia were not found to be predictors of chemotoxicity nor was time to tumor progression. |
| Cortellini et al., 2018[104] | Retrospective cohort | NSCLC,  stage IV | n = 81 | n = 28 by SMI  n = 23 by SMD | CT | Men: SMI at L3 < 43 cm^2^/m^2^ for those BMI < 25 kg/m^2^, < 53 cm^2^/m^2^ for those BMI ≥ 25kg/m^2^, SMD < 28.0 HU  Women: SMI at L3 < 41 cm^2^/m^2^, SMD < 23.8 HU | Chemotherapy | Low SMI was a significant predictor of shorter progression free survival, but not low SMD. A significant difference in hematological toxicities between patients with low and non-low SMI. |
| Cortellini et al., 2019[85] | Retrospective cohort | NSCLC,  stage IV | n = 23 | n = 9 | CT | Men: SMI at L3 < 43 cm^2^/m^2^ for those BMI < 25 kg/m^2^, < 53 cm^2^/m^2^ for those BMI ≥ 25kg/m^2^  Women: SMI at L3 < 41 cm^2^/m^2^ | Nivolumab | Prevalence of sarcopenia was 39.1%. There were trends towards longer median progression free survival and median overall survival in favor of patients without sarcopenia. However, no statistically significant differences were observed. |
| Martini et al.,  2020[105] | Retrospective cohort | Lung cancer,  stage I-IV | n = 234 | n = 78 | CT | Men: SMA at L3 < 135.8 cm^2^  Women: SMA at L3 < 97.0 cm^2^ | Surgery | Low SMA at L3 was independently predictive of higher chances of acute respiratory failure and 30-day mortality after pneumonectomy for lung cancer. |
| Takada et al., 2020[91] | Retrospective cohort | NSCLC,  stage IIIB-IV or recurrent | n = 103 | n = 51 | CT | Men: SMI at L3 < 25.63 cm^2^/m^2^  Women: SMI at L3 < 21.73cm^2^/m^2^ | Anti-PD-1 inhibitors | Prevalence of sarcopenia was 49.5%. Low SMI at L3 was an independent predictor of both progression free and overall survival. Disease control rate was also significantly lower in the sarcopenic group. No difference in response rate was seen between groups. |
| **Takenaka et al., 2021[106]** | | | | | |  |  |  |
| Cortellini et al., 2019[85] | Retrospective cohort | NSCLC,  stage IV | n = 23 | n - 9 | CT | Men: SMI at L3 < 43 cm^2^/m^2^ for those BMI < 25kg/m^2^, < 53 cm^2^/m^2^ for those BMI ≥ 25kg/m^2^  Women: SMI at L3 < 41 cm^2^/m^2^ | Nivolumab | There were trends towards longer median progression free survival and median overall survival in favor of patients without sarcopenia. However, no statistically significant differences were observed. |
| Nishioka et al., 2019[87] | Retrospective cohort | NSCLC,  stage III-IV or recurrent | n = 38 | n - 21 | CT | ≥ 10% loss of PMA at L2-3 between diagnosis and starting immunotherapy | Anti-PD-1 inhibitors | Patients with sarcopenia exhibited a significantly shorter median progression free survival than non-sarcopenia patients. |
| Shiroyama et al., 2019[88] | Retrospective cohort | NSCLC, pretreated, stage unspecified | n = 42 | n = 22 | CT | Men: PMI at L3 < 6.36 cm^2^/m^2^  Women: PMI at L3 < 3.92 cm^2^/m^2^ | Anti-PD-1 inhibitors | The prevalence of sarcopenia was 52.4%. Sarcopenia was significantly associated with poorer response rate and progression free survival. |
| Minami et al., 2020[89] | Retrospective cohort | NSCLC, pretreated, stage unspecified | n = 74 | n = 53 | CT | Men: PMI at L3 < 6.36 cm^2^/m^2^  Women: PMI at L3 < 3.92 cm^2^/m^2^ | Anti-PD-(L)1 inhibitors | There was no significant difference in overall survival and progression free survival according to PMI, intramuscular adipose tissue content or visceral-to-subcutaneous ratio. |
| Roch et al.,  2020[90] | Retrospective cohort | NSCLC,  stage IV or recurrent | n = 142 | n = 92 | CT | Men: SMI at L3 < 52·4 cm²/m²  Women: SMI at L3 < 38·5 cm²/m² | Anti-PD-(L)1 inhibitors | Patients with evolving sarcopenia had a shorter progression free survival and overall survival. |
| Takada et al., 2020[91] | Retrospective cohort | NSCLC,  stage IIIB-IV or recurrent | n = 103 | n = 51 | CT | Men: SMI at L3 < 25.63 cm^2^/m^2^  Women: SMI at L3 <21.73cm^2^/m^2^ | Anti-PD-1 inhibitors | Low SMI at L3 was an independent predictor of both progression free and overall survival. Disease control rate was also significantly lower in the sarcopenic group. No difference in response rate was seen between groups. |
| Tsukagoshi et al., 2020[92] | Retrospective cohort | NSCLC,  stage III-IV or recurrent | n = 30 | n = 13 | CT | Men: PMI at L3 < 6.36 cm^2^/m^2^  Women: PMI at L3 < 3.92 cm^2^/m^2^ | Nivolumab | There was no significant association between sarcopenia and immune-related adverse events. Muscle loss was significantly associated with fewer nivolumab cycles, poorer response rate, shorter progression-free survival, and median overall survival. |
| Nishioka et al., 2021[107] | Retrospective cohort | NSCLC,  stage III-IV | n = 156 | n = 47 by SMI  n = 80 by SMD | CT | Men: SMI at L3 < 43 cm^2^/m^2^ for those BMI < 25 kg/m^2^, < 53 cm^2^/m^2^ for those BMI ≥ 25 kg/m^2^  Women: SMI at L3 <41 cm^2^/m^2^,  Men/women: SMD < 41 HU for those BMI < 25 kg/m^2^, < 33 HU for those ≥ 25 kg/m^2^ | Anti-PD-(L)1 inhibitors | Patients with high muscle quality showed higher overall response rate and longer progression free survival durations. |
| **Surov et al., 2022[108]** | | | |  |  |  |  |  |
| Kim et al., 2016[103] | Retrospective cohort | SCLC, limited or extended | n = 186 | n = 128 | CT | Men: SMI at L3 < 55 cm2/m2  Women: SMI at L3 < 39 cm2/m2 | NA | Prevalence of sarcopenia was 68.8%. Sarcopenic patients had significantly higher neutrophil-lymphocyte ratios and CRP levels than non-sarcopenic patients. CRP independently predicted SMI and performance status. |
| Srdic et al., 2016[81] | Longitudinal | NSCLC,  stage IIIB-IV | n = 55 | n = 30 | CT | Men: SMI at L3 < 55 cm^2^/m^2^  Women: SMI at L3 < 39 cm^2^/m^2^ | Chemotherapy | Cachexia and sarcopenia were not found to be predictors of chemotoxicity nor was time to tumor progression. |
| Suzuki et al.,  2016[62] | Retrospective  cohort | NSCLC,  stage IA-IB | n = 90 | n = 38 | CT | Men: SMI at L3 < 43.75 cm^2^/m^2^  Women: SMI at L3 < 41.10cm^2^/m^2^ | Surgery | Patients with sarcopenia were more likely to have a low BMI. Multivariate analysis found that sarcopenia was a significant independent prognostic factor (HR: 7.09). |
| Naito et al., 2019[109] | Longitudinal | NSCLC,  Stage III-IV or recurrent | n = 30 | n = 20 | CT | Men: SMI at L3 < 43 cm^2^/m^2^ for those BMI < 25 kg/m^2^, < 53 cm^2^/m^2^ for those BMI ≥ 25 kg/m^2^  Women: SMI at L3 < 41 cm^2^/m^2^ | Chemotherapy | Change in muscle mass was significantly associated with change in grip strength and incremental shuttle walking distance. |
| Shoji et al., 2017[65] | Retrospective  cohort | NSCLC,  stage IA-IIA | n = 147 | n = 57 | CT | Men: SMI at L3 < 43.75 cm^2^/m^2^  Women: SMI at L3 < 41.10 cm^2^/m^2^ | Surgery +/- adjuvant chemotherapy | Pre-operative sarcopenia was significantly associated with poor survival (HR: 5.138, 95%CI: 2.305-11.676) and immune-nutritional parameters in early NSCLC. |
| Kim et al., 2017[73] | Retrospective cohort | NSCLC,  stage I-IV | n = 272 | n = 61 | CT | Men: SMI at L3 < 55 cm^2^/m^2^  Women: SMI at L3 < 39 cm^2^/m^2^ | Surgery | The prevalence of sarcopenia was 22.4%. No significant difference was observed between patients with or without sarcopenia in terms of ICU or hospital stay, post-operative complications, 3-year disease free survival or 3-year overall survival. |
| Cortellini et al., 2018[104] | Retrospective cohort | NSCLC,  stage IV | n = 81 | n = 28 by SMI  n = 23 by SMD | CT | Men: SMI at L3 < 43 cm^2^/m^2^ for those BMI < 25 kg/m^2^, < 53 cm^2^/m^2^ for those BMI ≥ 25kg/m^2^, SMD < 28.0 HU  Women: SMI at L3 < 41 cm^2^/m^2^, SMD < 23.8 HU | Chemotherapy | Low SMI was a significant predictor of shorter progression free survival, but not low SMD. A significant difference in hematological toxicities between patients with low and non-low SMI. |
| Recio-Bioles et al., 2018[110] | Longitudinal | NSCLC,  stage III-IV | n =37 | n = 5 | CT | Men: SMI at L3 < 52.4 cm^2^/m^2^  Women: SMI at L3 < 38.5 cm^2^/m^2^ |  | Sarcopenia was detected in 14.7% of patients; 20% had sarcopenic obesity. BMI correlation with SMI was weak. |
| Rossi et al., 2018[69] | Retrospective cohort | NSCLC,  stage IV | n = 33 | n = 20 | CT | Men: SMI at L3 ≤ 55 cm^2^/m^2^  Women: SMI at L3 ≤ 39 cm^2^/m^2^ | Gefitinib | Sarcopenia did not affect response to gefitinib treatment in EGFR mutated NSCLC patients, even if it is a bad prognostic indicator for overall survival. |
| Antoun et al.,  2019[111] | Cross-sectional, multicenter study | NSCLC,  Stage I-IV | n =312 | n= 167 | CT | Men: SMI at L3 < 43 cm^2^/m^2^ for those BMI < 25 kg/m^2^, < 53 cm^2^/m^2^ for those BMI ≥ 25 kg/m^2^  Women: SMI at L3 < 41 cm^2^/m^2^ | N/A | Sarcopenia was present in 66.7% of cancer cachexia group and 68.5% of pre‐cachexia patients. |
| Portal et al., 2019[112] | Retrospective cohort | NSCLC,  stage I-IV | n = 140 | n = 92 | CT | Men: SMI at L3 < 53 cm²/m²  Women: SMI at L3 < 41 cm²/m | N/A | Sarcopenia was significantly associated with mortality in univariate analysis but not in multivariate analysis. |
| Sun et al.,  2019[113] | Retrospective cohort | NSCLC,  stage I-II | n = 314 | n = 78 | CT | Men: SMI at L1 < 38 cm²/m²  Women: SMI at L1 < 29.6 cm²/m | Surgery | Low SMI at L1 was an independent predictor for worse overall survival. |
| Lee eet al.,  2020[114] | Retrospective cohort | NSCLC,  stage I-II | n =236 | n = 59 | CT | Men: psoas volume index at L3 < 71.31 cm^3^/m^3^  Women: psoas volume index at L3 < 51.87 cm^3^/m^3^ | Surgery | Low psoas volume index was associated with a higher rate of early post-operative complications in patients with early-stage NSCLC. |
| Roch et al.,  2020[90] | Retrospective cohort | NSCLC,  stage IV or recurrent | n = 142 | n = 92 | CT | Men: SMI at L3 < 52·4 cm²/m²  Women: SMI at L3 < 38·5 cm²/m² | Anti-PD-(L)1 inhibitors | Patients with evolving sarcopenia had a shorter progression free survival and overall survival. |
| Choi et al., 2021[115] | Retrospective cohort | NSCLC,  stage I | n = 440 | n = 246 | CT | Men: SMI at L3 < 55 cm^2^/m^2^  Women: SMI at L3 < 39 cm^2^/m^2^ | Surgery | Adipopenia was associated with reduced 5-year overall survival rate in early NSCLC and may indicate non-cancer-related death. |
| Nie et al., 2021[116] | Retrospective cohort | NSCLC,  stage | n = 35 | n = 24 | CT | Men: SMI at L3 < 52·4 cm²/m²  Women: SMI at L3 < 38·5 cm²/m² | Afatinib | Sarcopenic patients had a significantly higher rate of grade ≥ 2 diarrhea and toxicity-related dose reduction. Multivariate analysis showed that sarcopenia was an independent risk factor for dose reduction of afatinib. Both dose reduction and sarcopenia did not affect therapeutic efficacy. |
| **Voorn et al, 2022[117]** | | | | | | |  |  |
| Jagoe et al., 2001[25] | Cross-sectional | Lung cancer, stage I-IV | n = 60  (and 22 controls) | NA | BIA, grip strength, four skinfold method | NA | Surgery | Weight, grip strength and skin fold thickness were not significantly different between patients referred for lung cancer surgery and healthy controls. |
| Tsukioka et al., 2017[66] | Retrospective cohort | NSCLC, I | n = 215 | n =30 | CT | Men: SMI at L3 < 49 cm²/m²  Only male patients were included | Surgery | Sarcopenia was significantly associated with BMI, nutritional condition, serum CYFRA 21-1 level and pathological stage, but not with pre-operative respiratory function or performance status. The sarcopenia group had a significantly shorter median overall survival than the non-sarcopenia group. |
| Kim et al., 2017[73] | Retrospective cohort | NSCLC,  stage I-IV | n = 272 | n = 61 | CT | Men: SMI at L3 < 55 cm^2^/m^2^  Women: SMI at L3 < 39 cm^2^/m^2^ | Surgery | The prevalence of sarcopenia was 22.4%. No significant difference was observed between patients with or without sarcopenia in terms of ICU or hospital stay, post-operative complications, 3-year disease free survival or 3-year overall survival. |
| Nakamura et al., 2018[74] | Retrospective cohort | NSCLC,  stage I-III | n = 328 | n = 183 | CT | Men: PMI at L3 < 6.36 cm^2^/m^2^  Women: PMI at L3 < 3.92 cm^2^/m^2^ | Surgery | Sarcopenia was significantly related with increasing age, male sex, smoking habit, lower BMI, and post-operative major complication. Multivariate analysis found that sarcopenia was an independent unfavorable prognostic factor for survival. |
| Kawaguchi et al., 2019[97] | Retrospective cohort | NSCLC,  stage 0, I-III | n = 173 | n = 32 | CT | Men: PMI at L3 < 3.70 cm^2^/m^2^  Women: PMI at L3 < 2.50 cm^2^/m^2^ | Surgery | Sarcopenia was a strong and independent predictor for survival in elderly NSCLC patients. |
| Nakada et al., 2019[118] | Retrospective cohort | NSCLC,  stage I | n = 173 | n = 58 | CT | Men: PMI at L3 < 4.61 cm^2^/m^2^  Women: PMI at L3 < 3.26 cm^2^/m^2^ | Surgery | Sarcopenia was not significantly related to complications after complete single-lobe thoracoscopic lobectomies for clinical stage I NSCLC. |
| Madariaga et al., 2020[119] | Retrospective cohort | Lung cancer, stage I-IV | n = 130 | NA | CT | Thoracic SMA analyzed as a continuous variable | Surgery | Patients with high thoracic SMA experienced fewer overall and cardiopulmonary complications. Associations with ICU length of stay and hospital length of stay did not reach significance. |

ALM: appendicular lean mass, ATP: , BMI: body mass index, BIA: bioelectrical impedance analysis, CRP: C-reactive protein, CT: computed tomography, DEXA: dual-energy x-ray absorptiometry, DHA: docosahexaenoic acid, EGFR: epidermal growth factor receptor, EPA: eicosatetaenoic acid, HR: hazard ratio, HU: Hounsfield unit, IGF-1: insulin-like growth factor-1, L1/3: first/third lumbar vertebrae, NA: not available, NSCLC: non-small cell lung cancer, PMA: psoas muscle area, PMI: psoas muscle index, RCT: randomized control trial, SCLC: small cell lung cancer, SMA: skeletal muscle area, SMD: skeletal muscle density, SMI: skeletal muscle index, T5/8/12: fifth/eighth/twelfth thoracic vertebrae. Note: For reviews involving multiple cancer types, only studies focusing on lung cancer or incoporated in the lung cancer subgroup were presented in this table. Likewise, only studies that investigated sarcopenia were extracted from the systemic review by Voorn. et al.[117].

**References**

1. Shachar SS, Williams GR, Muss HB, Nishijima TF. Prognostic value of sarcopenia in adults with solid tumours: A meta-analysis and systematic review. Eur J Cancer. 2016;57:58-67.

2. Pamoukdjian F, Bouillet T, Lévy V, Soussan M, Zelek L, Paillaud E. Prevalence and predictive value of pre-therapeutic sarcopenia in cancer patients: A systematic review. Clin Nutr. 2018;37(4):1101-13.

3. Pereira MME, Queiroz MDSC, de Albuquerque NMC, Rodrigues J, Wiegert EVM, Calixto-Lima L, et al. The Prognostic Role of Phase Angle in Advanced Cancer Patients: A Systematic Review. Nutrition in Clinical Practice. 2018;33(6):813-24.

4. Aleixo GFP, Shachar SS, Nyrop KA, Muss HB, Battaglini CL, Williams GR. Bioelectrical Impedance Analysis for the Assessment of Sarcopenia in Patients with Cancer: A Systematic Review. Oncologist. 2020;25(2):170-82.

5. Aleixo GFP, Shachar SS, Nyrop KA, Muss HB, Malpica L, Williams GR. Myosteatosis and prognosis in cancer: Systematic review and meta-analysis. Crit Rev Oncol Hematol. 2020;145:102839.

6. Huiskamp LFJ, Chargi N, Devriese LA, May AM, Huitema ADR, de Bree R. The Predictive Value of Low Skeletal Muscle Mass Assessed on Cross-Sectional Imaging for Anti-Cancer Drug Toxicity: A Systematic Review and Meta-Analysis. J Clin Med. 2020;9(11).

7. Jang MK, Park C, Hong S, Li H, Rhee E, Doorenbos AZ. Skeletal Muscle Mass Change During Chemotherapy: A Systematic Review and Meta-analysis. Anticancer Res. 2020;40(5):2409-18.

8. Wiegert EVM, de Oliveira LC, Calixto-Lima L, Borges NA, Rodrigues J, da Mota e Silva Lopes MS, et al. Association between Low Muscle Mass and Survival in Incurable Cancer Patients: A Systematic Review. Nutrition. 2020;72.

9. Günther S, Trinkner P, Von Bergwelt M, Cordas Dos Santos D, Theurich S. SARCOPENIA AS BIOMARKER for IMMUNOTHERAPY OUTCOMES and IMMUNE-RELATED ADVERSE EVENTS - A SYSTEMATIC REVIEW and META-ANALYSIS. Journal for ImmunoTherapy of Cancer. 2021;9(SUPPL 1):A7.

10. Guzman-Prado Y, Ben Shimol J, Samson O. Sarcopenia and the risk of adverse events in patients treated with immune checkpoint inhibitors: a systematic review. Cancer Immunol Immunother. 2021;70(10):2771-80.

11. Hanna L, Nguo K, Furness K, Porter J, Huggins CE. Association between between skeletal muscle mass and quality of life in adults with cancer: A systematic review and meta-analysis. Asia-Pacific Journal of Clinical Oncology. 2021;17(SUPPL 9):170-1.

12. Li S, Wang T, Lai W, Zhang M, Cheng B, Wang S, et al. Prognostic impact of sarcopenia on immune-related adverse events in malignancies received immune checkpoint inhibitors: a systematic review and meta-analysis. Transl Cancer Res. 2021;10(12):5150-8.

13. Matthews L, Bates A, Wootton SA, Levett D. The use of bioelectrical impedance analysis to predict post-operative complications in adult patients having surgery for cancer: A systematic review. Clinical Nutrition. 2021;40(5):2914-22.

14. Surov A, Pech M, Gessner D, Mikusko M, Fischer T, Alter M, et al. Low skeletal muscle mass is a predictor of treatment related toxicity in oncologic patients. A meta-analysis. Clin Nutr. 2021;40(10):5298-310.

15. Tan H, Gao X, Li X, Huang Y, Cao Q, Wan T. Sarcopenia in Patients With Spinal Metastasis: A Systematic Review and Meta-Analysis of Retrospective Cohort Studies. Front Oncol. 2022;12:864501.

16. Bullock AF, Greenley SL, McKenzie GAG, Paton LW, Johnson MJ. Relationship between markers of malnutrition and clinical outcomes in older adults with cancer: systematic review, narrative synthesis and meta-analysis. European Journal of Clinical Nutrition. 2020;74(11):1519-35.

17. Payne C, Larkin PJ, McIlfatrick S, Dunwoody L, Gracey JH. Exercise and nutrition interventions in advanced lung cancer: A systematic review. Current Oncology. 2013;20(4):e321-e37.

18. Polański J, Chabowski M, Świątoniowska-Lonc N, Dudek K, Jankowska-Polańska B, Zabierowski J, et al. Relationship between Nutritional Status and Clinical Outcome in Patients Treated for Lung Cancer. Nutrients. 2021;13(10).

19. Zhang X, Tang T, Pang L, Sharma SV, Li R, Nyitray AG, et al. Malnutrition and overall survival in older adults with cancer: A systematic review and meta-analysis. J Geriatr Oncol. 2019;10(6):874-83.

20. Collins J, Noble S, Chester J, Coles B, Byrne A. The assessment and impact of sarcopenia in lung cancer: a systematic literature review. BMJ Open. 2014;4(1):e003697.

21. Hansell DT, Davies JW, Burns HJ. The relationship between resting energy expenditure and weight loss in benign and malignant disease. Ann Surg. 1986;203(3):240-5.

22. Fredrix EW, Soeters PB, Wouters EF, Deerenberg IM, von Meyenfeldt MF, Saris WH. Energy balance in relation to cancer cachexia. Clin Nutr. 1990;9(6):319-24.

23. Simons JP, Schols AM, Campfield LA, Wouters EF, Saris WH. Plasma concentration of total leptin and human lung-cancer-associated cachexia. Clin Sci (Lond). 1997;93(3):273-7.

24. Simons JP, Schols AM, Buurman WA, Wouters EF. Weight loss and low body cell mass in males with lung cancer: relationship with systemic inflammation, acute-phase response, resting energy expenditure, and catabolic and anabolic hormones. Clin Sci (Lond). 1999;97(2):215-23.

25. Jagoe RT, Goodship TH, Gibson GJ. Nutritional status of patients undergoing lung cancer operations. The Annals of thoracic surgery. 2001;71(3):929-35.

26. Jatoi A, Daly BD, Hughes VA, Dallal GE, Kehayias J, Roubenoff R. Do patients with nonmetastatic non-small cell lung cancer demonstrate altered resting energy expenditure? Ann Thorac Surg. 2001;72(2):348-51.

27. McMillan DC, Watson WS, O'Gorman P, Preston T, Scott HR, McArdle CS. Albumin concentrations are primarily determined by the body cell mass and the systemic inflammatory response in cancer patients with weight loss. Nutr Cancer. 2001;39(2):210-3.

28. Scott HR, McMillan DC, Watson WS, Milroy R, McArdle CS. Longitudinal study of resting energy expenditure, body cell mass and the inflammatory response in male patients with non-small cell lung cancer. Lung Cancer. 2001;32(3):307-12.

29. Agteresch HJ, Rietveld T, Kerkhofs LG, van den Berg JW, Wilson JH, Dagnelie PC. Beneficial effects of adenosine triphosphate on nutritional status in advanced lung cancer patients: a randomized clinical trial. J Clin Oncol. 2002;20(2):371-8.

30. Crown AL, Cottle K, Lightman SL, Falk S, Mohamed-Ali V, Armstrong L, et al. What is the role of the insulin-like growth factor system in the pathophysiology of cancer cachexia, and how is it regulated? Clin Endocrinol (Oxf). 2002;56(6):723-33.

31. Jagoe RT, Redfern CP, Roberts RG, Gibson GJ, Goodship TH. Skeletal muscle mRNA levels for cathepsin B, but not components of the ubiquitin-proteasome pathway, are increased in patients with lung cancer referred for thoracotomy. Clin Sci (Lond). 2002;102(3):353-61.

32. Harvie MN, Campbell IT, Thatcher N, Baildam A. Changes in body composition in men and women with advanced nonsmall cell lung cancer (NSCLC) undergoing chemotherapy. J Hum Nutr Diet. 2003;16(5):323-6.

33. Sarhill N, Mahmoud F, Walsh D, Nelson KA, Komurcu S, Davis M, et al. Evaluation of nutritional status in advanced metastatic cancer. Support Care Cancer. 2003;11(10):652-9.

34. Bauer JD, Capra S. Nutrition intervention improves outcomes in patients with cancer cachexia receiving chemotherapy--a pilot study. Support Care Cancer. 2005;13(4):270-4.

35. Harvie MN, Howell A, Thatcher N, Baildam A, Campbell I. Energy balance in patients with advanced NSCLC, metastatic melanoma and metastatic breast cancer receiving chemotherapy–a longitudinal study. British journal of cancer. 2005;92(4):673-80.

36. Fearon KC, Barber MD, Moses AG, Ahmedzai SH, Taylor GS, Tisdale MJ, et al. Double-blind, placebo-controlled, randomized study of eicosapentaenoic acid diester in patients with cancer cachexia. J Clin Oncol. 2006;24(21):3401-7.

37. Wieland BM, Stewart GD, Skipworth RJ, Sangster K, Fearon KC, Ross JA, et al. Is there a human homologue to the murine proteolysis-inducing factor? Clin Cancer Res. 2007;13(17):4984-92.

38. Bovio G, Bettaglio R, Bonetti G, Miotti D, Verni P. Evaluation of nutritional status and dietary intake in patients with advanced cancer on palliative care. Minerva gastroenterologica e dietologica. 2008;54(3):243-50.

39. Prado CM, Lieffers JR, McCargar LJ, Reiman T, Sawyer MB, Martin L, et al. Prevalence and clinical implications of sarcopenic obesity in patients with solid tumours of the respiratory and gastrointestinal tracts: a population-based study. The lancet oncology. 2008;9(7):629-35.

40. Tozer RG, Tai P, Falconer W, Ducruet T, Karabadjian A, Bounous G, et al. Cysteine-rich protein reverses weight loss in lung cancer patients receiving chemotherapy or radiotherapy. Antioxid Redox Signal. 2008;10(2):395-402.

41. Trutschnigg B, Kilgour RD, Reinglas J, Rosenthall L, Hornby L, Morais JA, et al. Precision and reliability of strength (Jamar vs. Biodex handgrip) and body composition (dual-energy X-ray absorptiometry vs. bioimpedance analysis) measurements in advanced cancer patients. Applied Physiology, Nutrition, and Metabolism. 2008;33(6):1232-9.

42. Beijer S, Hupperets PS, van den Borne BE, Eussen SR, van Henten AM, van den Beuken-van Everdingen M, et al. Effect of adenosine 5'-triphosphate infusions on the nutritional status and survival of preterminal cancer patients. Anticancer Drugs. 2009;20(7):625-33.

43. Vigano A, Trutschnigg B, Kilgour RD, Hamel N, Hornby L, Lucar E, et al. Relationship between angiotensin-converting enzyme gene polymorphism and body composition, functional performance, and blood biomarkers in advanced cancer patients. Clin Cancer Res. 2009;15(7):2442-7.

44. Baracos VE, Reiman T, Mourtzakis M, Gioulbasanis I, Antoun S. Body composition in patients with non− small cell lung cancer: A contemporary view of cancer cachexia with the use of computed tomography image analysis. The American journal of clinical nutrition. 2010;91(4):1133S-7S.

45. Kilgour RD, Vigano A, Trutschnigg B, Hornby L, Lucar E, Bacon SL, et al. Cancer-related fatigue: the impact of skeletal muscle mass and strength in patients with advanced cancer. J Cachexia Sarcopenia Muscle. 2010;1(2):177-85.

46. Murphy RA, Mourtzakis M, Chu QS, Reiman T, Mazurak VC. Skeletal muscle depletion is associated with reduced plasma (n-3) fatty acids in non-small cell lung cancer patients. J Nutr. 2010;140(9):1602-6.

47. Murphy RA, Mourtzakis M, Chu QS, Baracos VE, Reiman T, Mazurak VC. Nutritional intervention with fish oil provides a benefit over standard of care for weight and skeletal muscle mass in patients with nonsmall cell lung cancer receiving chemotherapy. Cancer. 2011;117(8):1775-82.

48. Martínez-Hernández PL, Hernanz-Macías Á, Gómez-Candela C, Grande-Aragón C, Feliu-Batlle J, Castro-Carpeño J, et al. Serum interleukin-15 levels in cancer patients with cachexia. Oncol Rep. 2012;28(4):1443-52.

49. Op den Kamp CM, Langen RC, Minnaard R, Kelders MC, Snepvangers FJ, Hesselink MK, et al. Pre-cachexia in patients with stages I-III non-small cell lung cancer: systemic inflammation and functional impairment without activation of skeletal muscle ubiquitin proteasome system. Lung Cancer. 2012;76(1):112-7.

50. Peddle-McIntyre CJ, Bell G, Fenton D, McCargar L, Courneya KS. Feasibility and preliminary efficacy of progressive resistance exercise training in lung cancer survivors. Lung Cancer. 2012;75(1):126-32.

51. Winter A, MacAdams J, Chevalier S. Normal protein anabolic response to hyperaminoacidemia in insulin-resistant patients with lung cancer cachexia. Clin Nutr. 2012;31(5):765-73.

52. Martin L, Birdsell L, MacDonald N, Reiman T, Clandinin MT, McCargar LJ, et al. Cancer cachexia in the age of obesity: skeletal muscle depletion is a powerful prognostic factor, independent of body mass index. Journal of clinical oncology. 2013;31(12):1539-47.

53. Op den Kamp CM, Langen RC, Snepvangers FJ, de Theije CC, Schellekens JM, Laugs F, et al. Nuclear transcription factor κ B activation and protein turnover adaptations in skeletal muscle of patients with progressive stages of lung cancer cachexia. The American journal of clinical nutrition. 2013;98(3):738-48.

54. Prado CM, Sawyer MB, Ghosh S, Lieffers JR, Esfandiari N, Antoun S, et al. Central tenet of cancer cachexia therapy: do patients with advanced cancer have exploitable anabolic potential? Am J Clin Nutr. 2013;98(4):1012-9.

55. Buentzel J, Heinz J, Bleckmann A, Bauer C, Röver C, Bohnenberger H, et al. Sarcopenia as Prognostic Factor in Lung Cancer Patients: A Systematic Review and Meta-analysis. Anticancer Res. 2019;39(9):4603-12.

56. Gupta D, Lammersfeld CA, Vashi PG, King J, Dahlk SL, Grutsch JF, et al. Bioelectrical impedance phase angle in clinical practice: implications for prognosis in stage IIIB and IV non-small cell lung cancer. BMC cancer. 2009;9(1):1-6.

57. Sánchez-Lara K, Turcott JG, Juárez E, Guevara P, Núñez-Valencia C, Oñate-Ocaña LF, et al. Association of nutrition parameters including bioelectrical impedance and systemic inflammatory response with quality of life and prognosis in patients with advanced non-small-cell lung cancer: a prospective study. Nutrition and cancer. 2012;64(4):526-34.

58. Kim EY, Kim YS, Park I, Ahn HK, Cho EK, Jeong YM. Prognostic Significance of CT-Determined Sarcopenia in Patients with Small-Cell Lung Cancer. Journal of Thoracic Oncology. 2015;10(12):1795-9.

59. Kimura M, Naito T, Kenmotsu H, Taira T, Wakuda K, Oyakawa T, et al. Prognostic impact of cancer cachexia in patients with advanced non-small cell lung cancer. Supportive Care in Cancer. 2015;23(6):1699-708.

60. Stene GB, Helbostad JL, Amundsen T, Sørhaug S, Hjelde H, Kaasa S, et al. Changes in skeletal muscle mass during palliative chemotherapy in patients with advanced lung cancer. Acta Oncologica. 2015;54(3):340-8.

61. Sjøblom B, Grønberg BH, Wentzel-Larsen T, Baracos VE, Hjermstad MJ, Aass N, et al. Skeletal muscle radiodensity is prognostic for survival in patients with advanced non-small cell lung cancer. Clinical Nutrition. 2016;35(6):1386-93.

62. Suzuki Y, Okamoto T, Fujishita T, Katsura M, Akamine T, Takamori S, et al. Clinical implications of sarcopenia in patients undergoing complete resection for early non-small cell lung cancer. Lung cancer. 2016;101:92-7.

63. Bowden J, Williams L, Simms A, Price A, Campbell S, Fallon M, et al. Prediction of 90 day and overall survival after chemoradiotherapy for lung cancer: role of performance status and body composition. Clinical Oncology. 2017;29(9):576-84.

64. Kinsey CM, San José Estépar R, Van der Velden J, Cole BF, Christiani DC, Washko GR. Lower Pectoralis Muscle Area Is Associated with a Worse Overall Survival in Non–Small Cell Lung CancerPectoralis Muscle Area and NSCLC Survival. Cancer Epidemiology, Biomarkers & Prevention. 2017;26(1):38-43.

65. Shoji F, Matsubara T, Kozuma Y, Haratake N, Akamine T, Takamori S, et al. Relationship between preoperative sarcopenia status and immuno-nutritional parameters in patients with early-stage non-small cell lung cancer. Anticancer research. 2017;37(12):6997-7003.

66. Tsukioka T, Nishiyama N, Izumi N, Mizuguchi S, Komatsu H, Okada S, et al. Sarcopenia is a novel poor prognostic factor in male patients with pathological Stage I non-small cell lung cancer. Japanese Journal of Clinical Oncology. 2017;47(4):363-8.

67. Chambard L, Girard N, Ollier E, Rousseau J-C, Duboeuf F, Carlier M-C, et al. Bone, muscle, and metabolic parameters predict survival in patients with synchronous bone metastases from lung cancers. Bone. 2018;108:202-9.

68. Mitsuyoshi T, Matsuo Y, Itou H, Shintani T, Iizuka Y, Kim YH, et al. Evaluation of a prognostic scoring system based on the systemic inflammatory and nutritional status of patients with locally advanced non-small-cell lung cancer treated with chemoradiotherapy. Journal of Radiation Research. 2018;59(1):50-7.

69. Rossi S, Di Noia V, Tonetti L, Strippoli A, Basso M, Schinzari G, et al. Does sarcopenia affect outcome in patients with non-small-cell lung cancer harboring EGFR mutations? Future Oncology. 2018;14(10):919-26.

70. Takamori S, Toyokawa G, Okamoto T, Shimokawa M, Kinoshita F, Kozuma Y, et al. Clinical Impact and Risk Factors for Skeletal Muscle Loss After Complete Resection of Early Non-small Cell Lung Cancer. Ann Surg Oncol. 2018;25(5):1229-36.

71. Deng HY, Hou L, Zha P, Huang KL, Peng L. Sarcopenia is an independent unfavorable prognostic factor of non-small cell lung cancer after surgical resection: A comprehensive systematic review and meta-analysis. Eur J Surg Oncol. 2019;45(5):728-35.

72. Hervochon R, Bobbio A, Guinet C, Mansuet-Lupo A, Rabbat A, Régnard J-F, et al. Body mass index and total psoas area affect outcomes in patients undergoing pneumonectomy for cancer. The Annals of Thoracic Surgery. 2017;103(1):287-95.

73. Kim EY, Lee HY, Kim KW, Lee JI, Kim YS, Choi WJ, et al. Preoperative Computed Tomography–Determined Sarcopenia and Postoperative Outcome After Surgery for Non-Small Cell Lung Cancer. Scandinavian Journal of Surgery. 2017;107(3):244-51.

74. Nakamura R, Inage Y, Tobita R, Yoneyama S, Numata T, Ota K, et al. Sarcopenia in resected NSCLC: effect on postoperative outcomes. Journal of Thoracic Oncology. 2018;13(7):895-903.

75. Nishimura JM, Ansari AZ, D'Souza DM, Moffatt-Bruce SD, Merritt RE, Kneuertz PJ. Computed Tomography-Assessed Skeletal Muscle Mass as a Predictor of Outcomes in Lung Cancer Surgery. Ann Thorac Surg. 2019;108(5):1555-64.

76. Fintelmann FJ, Troschel FM, Mario J, Chretien YR, Knoll SJ, Muniappan A, et al. Thoracic skeletal muscle is associated with adverse outcomes after lobectomy for lung cancer. The Annals of Thoracic Surgery. 2018;105(5):1507-15.

77. Miller JA, Harris K, Roche C, Dhillon S, Battoo A, Demmy T, et al. Sarcopenia is a predictor of outcomes after lobectomy. Journal of thoracic disease. 2018;10(1):432-40.

78. Troschel FM, Kuklinski MW, Knoll SJ, Best TD, Muniappan A, Gaissert HA, et al. Preoperative thoracic muscle area on computed tomography predicts long-term survival following pneumonectomy for lung cancer. Interactive CardioVascular and Thoracic Surgery. 2019;28(4):542-9.

79. Yang M, Shen Y, Tan L, Li W. Prognostic Value of Sarcopenia in Lung Cancer: A Systematic Review and Meta-analysis. Chest. 2019;156(1):101-11.

80. Go S-I, Park MJ, Song H-N, Kang MH, Park HJ, Jeon KN, et al. Sarcopenia and inflammation are independent predictors of survival in male patients newly diagnosed with small cell lung cancer. Supportive care in cancer. 2016;24(5):2075-84.

81. Srdic D, Plestina S, Sverko-Peternac A, Nikolac N, Simundic A-M, Samarzija M. Cancer cachexia, sarcopenia and biochemical markers in patients with advanced non-small cell lung cancer—chemotherapy toxicity and prognostic value. Supportive care in cancer. 2016;24(11):4495-502.

82. Matsuo Y, Mitsuyoshi T, Shintani T, Iizuka Y, Mizowaki T. Impact of low skeletal muscle mass on non-lung cancer mortality after stereotactic body radiotherapy for patients with stage I non-small cell lung cancer. Journal of geriatric oncology. 2018;9(6):589-93.

83. Wang J, Cao L, Xu S. Sarcopenia affects clinical efficacy of immune checkpoint inhibitors in non-small cell lung cancer patients: A systematic review and meta-analysis. Int Immunopharmacol. 2020;88:106907.

84. Strulov Shachar S, Fried R, Shafran I, Moskovitz MT, Williams GR, Bar-Sela G, et al. Body composition as predictor of toxicity and outcomes in patients with metastatic non-small cell lung cancer (mNSCLC) receiving nivolumab (Nivo). American Society of Clinical Oncology; 2018.

85. Cortellini A, Verna L, Porzio G, Bozzetti F, Palumbo P, Masciocchi C, et al. Predictive value of skeletal muscle mass for immunotherapy with nivolumab in non‐small cell lung cancer patients: a “hypothesis‐generator” preliminary report. Thoracic cancer. 2019;10(2):347-51.

86. Nigro E, Perrotta F, Scialò F, D’Agnano V, Mallardo M, Bianco A, et al. Food, Nutrition, Physical Activity and Microbiota: Which Impact on Lung Cancer? International Journal of Environmental Research and Public Health. 2021;18(5):2399.

87. Nishioka N, Uchino J, Hirai S, Katayama Y, Yoshimura A, Okura N, et al. Association of sarcopenia with and efficacy of anti-PD-1/PD-L1 therapy in non-small-cell lung cancer. Journal of clinical medicine. 2019;8(4):450.

88. Shiroyama T, Nagatomo I, Koyama S, Hirata H, Nishida S, Miyake K, et al. Impact of sarcopenia in patients with advanced non–small cell lung cancer treated with PD-1 inhibitors: A preliminary retrospective study. Scientific reports. 2019;9(1):1-7.

89. Minami S, Ihara S, Tanaka T, Komuta K. Sarcopenia and visceral adiposity did not affect efficacy of immune-checkpoint inhibitor monotherapy for pretreated patients with advanced non-small cell lung cancer. World journal of oncology. 2020;11(1):9.

90. Roch B, Coffy A, Jean-Baptiste S, Palaysi E, Daures J-P, Pujol J-L, et al. Cachexia-sarcopenia as a determinant of disease control rate and survival in non-small lung cancer patients receiving immune-checkpoint inhibitors. Lung Cancer. 2020;143:19-26.

91. Takada K, Yoneshima Y, Tanaka K, Okamoto I, Shimokawa M, Wakasu S, et al. Clinical impact of skeletal muscle area in patients with non-small cell lung cancer treated with anti-PD-1 inhibitors. Journal of Cancer Research and Clinical Oncology. 2020;146(5):1217-25.

92. Tsukagoshi M, Yokobori T, Yajima T, Maeno T, Shimizu K, Mogi A, et al. Skeletal muscle mass predicts the outcome of nivolumab treatment for non-small cell lung cancer. Medicine. 2020;99(7).

93. Au PC, Li HL, Lee GK, Li GH, Chan M, Cheung BM, et al. Sarcopenia and mortality in cancer: A meta-analysis. Osteoporos Sarcopenia. 2021;7(Suppl 1):S28-s33.

94. Deng HY, Chen ZJ, Qiu XM, Zhu DX, Tang XJ, Zhou Q. Sarcopenia and prognosis of advanced cancer patients receiving immune checkpoint inhibitors: A comprehensive systematic review and meta-analysis. Nutrition. 2021;90:111345.

95. Kawaguchi Y, Hanaoka J, Ohshio Y, Okamoto K, Kaku R, Hayashi K, et al. Does sarcopenia affect postoperative short- and long-term outcomes in patients with lung cancer?-a systematic review and meta-analysis. J Thorac Dis. 2021;13(3):1358-69.

96. Tsukioka T, Izumi N, Mizuguchi S, Kyukwang C, Komatsu H, Toda M, et al. Positive correlation between sarcopenia and elevation of neutrophil/lymphocyte ratio in pathological stage IIIA (N2-positive) non-small cell lung cancer patients. Gen Thorac Cardiovasc Surg. 2018;66(12):716-22.

97. Kawaguchi Y, Hanaoka J, Ohshio Y, Okamoto K, Kaku R, Hayashi K, et al. Sarcopenia predicts poor postoperative outcome in elderly patients with lung cancer. General Thoracic and Cardiovascular Surgery. 2019;67(11):949-54.

98. Icard P, Schussler O, Loi M, Bobbio A, Mansuet Lupo A, Wislez M, et al. Pre-disease and pre-surgery BMI, weight loss and sarcopenia impact survival of resected lung cancer independently of tumor stage. Cancers. 2020;12(2):266.

99. Ozeki N, Kawaguchi K, Fukui T, Nakamura S, Hakiri S, Mori S, et al. Psoas muscle mass in patients undergoing lung cancer surgery: a prognostic difference between squamous cell carcinoma and adenocarcinoma. Int J Clin Oncol. 2020;25(5):876-84.

100. Shinohara S, Otsuki R, Kobayashi K, Sugaya M, Matsuo M, Nakagawa M. Impact of sarcopenia on surgical outcomes in non-small cell lung cancer. Annals of surgical oncology. 2020;27(7):2427-35.

101. Lee D, Kim NW, Kim JY, Lee JH, Noh JH, Lee H, et al. Sarcopenia's Prognostic Impact on Patients Treated with Immune Checkpoint Inhibitors: A Systematic Review and Meta-Analysis. J Clin Med. 2021;10(22).

102. McGovern J, Dolan RD, Horgan PG, Laird BJ, McMillan DC. Computed tomography-defined low skeletal muscle index and density in cancer patients: observations from a systematic review. J Cachexia Sarcopenia Muscle. 2021;12(6):1408-17.

103. Kim EY, Kim YS, Seo J-Y, Park I, Ahn HK, Jeong YM, et al. The relationship between sarcopenia and systemic inflammatory response for cancer cachexia in small cell lung cancer. PloS one. 2016;11(8):e0161125.

104. Cortellini A, Palumbo P, Porzio G, Verna L, Giordano AV, Masciocchi C, et al. Single‐institution study of correlations between skeletal muscle mass, its density, and clinical outcomes in non‐small cell lung cancer patients treated with first‐line chemotherapy. Thoracic Cancer. 2018;9(12):1623-30.

105. Martini K, Chassagnon G, Fournel L, Prieto M, Hoang-Thi T-N, Halm N, et al. Sarcopenia as independent risk factor of postpneumonectomy respiratory failure, ARDS and mortality. Lung Cancer. 2020;149:130-6.

106. Takenaka Y, Takemoto N, Oya R, Inohara H. Prognostic impact of sarcopenia in patients with head and neck cancer treated with surgery or radiation: A meta-analysis. PLoS One. 2021;16(10):e0259288.

107. Nishioka N, Naito T, Notsu A, Mori K, Kodama H, Miyawaki E, et al. Unfavorable impact of decreased muscle quality on the efficacy of immunotherapy for advanced non‐small cell lung cancer. Cancer medicine. 2021;10(1):247-56.

108. Surov A, Wienke A. Prevalence of sarcopenia in patients with solid tumors: A meta-analysis based on 81,814 patients. JPEN J Parenter Enteral Nutr. 2022.

109. Naito T, Okayama T, Aoyama T, Ohashi T, Masuda Y, Kimura M, et al. Skeletal muscle depletion during chemotherapy has a large impact on physical function in elderly Japanese patients with advanced non-small-cell lung cancer. BMC Cancer. 2017;17(1):571.

110. Recio-Boiles A, Galeas JN, Goldwasser B, Sanchez K, Man LM, Gentzler RD, et al. Enhancing evaluation of sarcopenia in patients with non-small cell lung cancer (NSCLC) by assessing skeletal muscle index (SMI) at the first lumbar (L1) level on routine chest computed tomography (CT). Supportive Care in Cancer. 2018;26(7):2353-9.

111. Antoun S, Morel H, Souquet PJ, Surmont V, Planchard D, Bonnetain F, et al. Staging of nutrition disorders in non-small-cell lung cancer patients: utility of skeletal muscle mass assessment. J Cachexia Sarcopenia Muscle. 2019;10(4):782-93.

112. Portal D, Hofstetter L, Eshed I, Dan-Lantsman C, Sella T, Urban D, et al. L3 skeletal muscle index (L3SMI) is a surrogate marker of sarcopenia and frailty in non-small cell lung cancer patients. Cancer management and research. 2019;11:2579.

113. Sun C, Anraku M, Karasaki T, Kuwano H, Nagayama K, Nitadori J-I, et al. Low truncal muscle area on chest computed tomography: a poor prognostic factor for the cure of early-stage non-small-cell lung cancer. European Journal of Cardio-Thoracic Surgery. 2019;55(3):414-20.

114. Lee J, Moon SW, Choi JS, Hyun K, Moon YK, Moon MH. Impact of Sarcopenia on Early Postoperative Complications in Early-Stage Non-Small-Cell Lung Cancer. Korean J Thorac Cardiovasc Surg. 2020;53(3):93-103.

115. Choi H, Park YS, Na KJ, Park S, Park IK, Kang CH, et al. Association of Adipopenia at Preoperative PET/CT with Mortality in Stage I Non-Small Cell Lung Cancer. Radiology. 2021;301(3):645-53.

116. Nie X, Zhang P, Gao JY, Cheng G, Liu W, Li L. Sarcopenia as a predictor of initial administration dose of afatinib in patients with advanced non‐small cell lung cancer. Thoracic Cancer. 2021;12(12):1824-30.

117. Voorn MJJ, Beukers K, Trepels CMM, Bootsma GP, Bongers BC, Janssen-Heijnen MLG. Associations between pretreatment nutritional assessments and treatment complications in patients with stage I-III non-small cell lung cancer: A systematic review. Clin Nutr ESPEN. 2022;47:152-62.

118. Nakada T, Noda Y, Kato D, Shibasaki T, Mori S, Asano H, et al. Risk factors and cancer recurrence associated with postoperative complications after thoracoscopic lobectomy for clinical stage I non‐small cell lung cancer. Thoracic Cancer. 2019;10(10):1945-52.

119. Madariaga MLL, Troschel FM, Best TD, Knoll SJ, Gaissert HA, Fintelmann FJ. Low thoracic skeletal muscle area predicts morbidity after pneumonectomy for lung cancer. The Annals of Thoracic Surgery. 2020;109(3):907-13.
